# Early knapping techniques do not necessitate cultural transmission

**DOI:** 10.1126/sciadv.abo2894

**Published:** 2022-07-06

**Authors:** William D. Snyder, Jonathan S. Reeves, Claudio Tennie

**Affiliations:** 1 Department of Early Prehistory and Quaternary Ecology, Eberhard Karls University of Tübingen, Tübingen, Germany.; 2 Technological Primates Research Group, Max Planck Institute for Evolutionary Anthropology, Leipzig, Germany.; 3 Department of Human Evolution, Max Planck Institute for Evolutionary Anthropology, Leipzig, Germany.

## Abstract

Early stone tool production, or knapping, techniques are claimed to be the earliest evidence for cultural transmission in the human lineage. Previous experimental studies have trained human participants to knap in conditions involving opportunities for cultural transmission. Subsequent knapping was then interpreted as evidence for a necessity of the provided cultural transmission opportunities for these techniques. However, a valid necessity claim requires showing that individual learning alone cannot lead to early knapping techniques. Here, we tested human participants (*N* = 28) in cultural isolation for the individual learning of early knapping techniques by providing them with relevant raw materials and a puzzle task as motivation. Twenty-five participants were technique naïve according to posttest questionnaires, yet they individually learned early knapping techniques, therewith producing and using core and flake tools. Early knapping techniques thus do not necessitate cultural transmission of know-how and could likewise have been individually derived among premodern hominins.

## Teaser

The early stone tool record is not unequivocal evidence for human-like cultural transmission in premodern hominins.

## INTRODUCTION

Cumulative culture of know-how defines the human niche (*1*–*4*). The products and cognition resulting from this special type of culture enable the adaptive success of the human species (*1*, *2*). This type of culture requires particular social learning mechanisms, namely, those that allow know-how information (Supplementary Text) (*3*, *5*) to be passed on intact and thereby to accumulate across generations until the accumulated know-how becomes impossible for single individuals to reinnovate (*4*, *6*). Despite its central importance, the origin of this type of cumulative culture within the human lineage is not well understood. Cumulative culture of know-how is argued to have emerged as early as the Oldowan industry (*7*–*11*), a stone tool technology that first appeared around 2.6 million years ago (*12*, *13*).

**Fig. 1.**
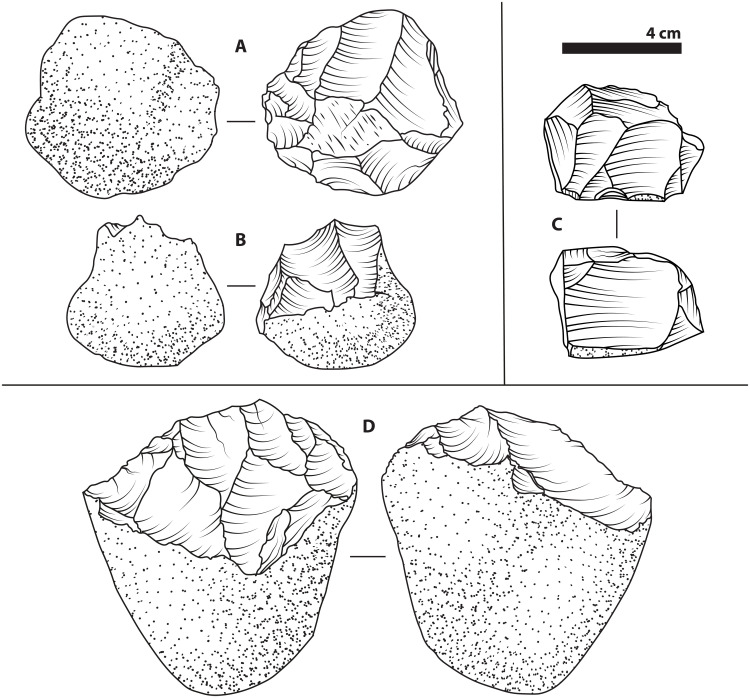
Oldowan technology. Illustrations based on various Oldowan-context core artifacts of differing reduction sequences and raw materials. The oldest, with an age of approximately 2.6 to 2.5 million years (Ma), are from Gona, Ethiopia (*73*) and include a radial core (**A**) and a unifacial chopper (**B**). An example of a multifacial core (**C**) comes from the site of Melka Kunture, Ethiopia, where premodern hominins exploited obsidian (volcanic glass) as a raw material, starting around 1.7 Ma (*55*). The final example is a bifacial core (**D**) from Hadar, Ethiopia and dated to approximately 2.3 Ma (*74*). In accordance with archaeological convention, solid lines are used for the outlines of scars (where flakes have been removed from a core surface), curved, incomplete lines represent the ripples from conchoidal fracturing of the knappable material, and dotted areas represent cortex (the unaltered, original surface of the raw material).

The central component of Oldowan technology was the use of percussion (e.g., with a hammerstone, anvil, or both) to create sharp edges on stone, typically via a mechanical process known as conchoidal fracture (*12*, *14*, *15*). The conchoidal fracture of materials such as flint, basalt, and glass, among others, leaves physical traces on the transformed materials that can be distinguished from the outcomes of other, importantly, less evolutionarily important, fracturing processes. The consequence of conchoidal fracture is the removal of “flakes,” which are visually identifiable by the presence of physical features such as a bulb of percussion, platform, and ripples (*12*, *15*). The repetition of this process within and across individuals resulted in assemblages of cores (Fig. 1) and detached pieces (thus, a “simple” core and flake technology) (*14*). Primarily, the flakes, but potentially also some cores, would have been used as cutting tools for butchering and other foraging tasks (*12*, *14*, *16*). This fundamental technological character (i.e., fracturing stone for the efficient production of cutting edge) not only is the basis of the Oldowan but also has appeared globally and in later periods up until the present. In this sense, the Oldowan and similar, later technologies from outside Africa have been labeled as Mode 1 industries, although even further classifications exist (*17*, *18*).

The technical know-how underlying Oldowan (and later Mode 1) artifacts, despite their comparative technological simplicity (*19*–*21*), is claimed to have already been maintained by the same types of social learning used by living modern humans, specifically, mechanisms able to transmit know-how that has culturally evolved beyond the individual reach (*7*–*11*, *22*, *23*). The main empirical basis for the claim that Oldowan artifacts provide evidence for such know-how copying skills is knapping experiments with human participants (*10*, *22*–*24*). These experiments showed a correlation between opportunities to learn how to knap via one or more of the social learning mechanisms (with models ranging from finished artifact forms to action demonstrations) known to be able to transmit know-how (*25*) and improved knapping performances. These improved knapping performances under varied social learning conditions were then typically interpreted to infer that special learning mechanisms able to transmit know-how were necessary for learning how to knap and for creating artifacts resembling those from the Oldowan. These conclusions for modern humans were then extended to Oldowan tool production by extinct hominins, claiming that premodern hominins would have also required the same, special types of social learning mechanisms to reproduce knapping techniques and outcomes (*10*, *22*–*24*).

In addition, previous studies have trained participants how to knap in abstraction from the inferred ultimate purpose of these artifacts, namely, tool use for extractive foraging and other tasks. In prehistory, expedient, least-effort toolmaking (*14*, *26*) is believed to have occurred when needed, e.g., to access nutrients. Instead, individuals in these knapping studies have been tested with the explicit, isolated goal of learning how to knap (with monetary compensation and personal interest in learning the skill being the main motivators) (*27*, *28*). Only very rarely have such experiments connected knapping with the goal of making usable tools, as in some studies where there was the provision of materials for the minimal testing of the sharpness of flakes (*29*–*31*). Consequently, earlier knapping studies potentially suffered from a lack of ecological relevance regarding tool use. Meanwhile, nonhuman knapping studies have solved this problem via the inclusion of baited puzzle boxes in testing [a simulation of extractive foraging, pioneered by Wright (*32*)]. Using such puzzle boxes creates a motivation for the production and subsequent use of cutting tools, without having to solely rely on social paradigms to motivate skill learning.

While earlier human knapping studies have clearly shown a correlation between improvement in some aspects of knapping and special types of social learning (although patterns across studies are often contradictory) (*10*, *22*, *23*, *33*), no study has tested whether cultural transmission of know-how is necessary. For example, it might be that various social learning mechanisms, as specifically expressed in modern humans, may merely speed up individual learning of knapping (some may even do so faster than others). This is an important caveat, as there are hypotheses, which posit that the knapping techniques from before and during the Oldowan (hereafter, early knapping techniques) should have been expressible by individuals in the full absence of social learning mechanisms able to directly transmit know-how (*34*, *35*). Under this account, some other types of social learning were likely still at play (e.g., the social transmission of know-where and know-what) but which only regulated the frequencies of such individual know-how reinnovations (*36*), creating “minimal cultures” (*37*, *38*). If true, the Oldowan would not mark the beginnings of modern human-like cumulative culture after all.

Earlier human knapping studies have not tested for the possibility of individual reinnovation of early knapping techniques (*34*, *35*). To test whether early knapping techniques can be arrived at individually, the development of knapping skill must be examined in cultural isolation from the underlying know-how. Said cultural isolation can be achieved by the “baseline test method” in which knapping technique know-how is made culturally inaccessible to the test participants. By test design, any type of target know-how transmission, be it via language, demonstrations, reverse engineering, or some other medium, must be strictly removed from the test situation (including before and during the experiment). This type of experimental paradigm is otherwise known as an island test (*34*, *38*). Island tests are the methodological benchmark for assessing the possibility of spontaneous and individual abilities to develop target know-how, such as early knapping techniques, without any opportunities for cultural transmission of their know-how (*34*, *38*).

**Fig. 2.**
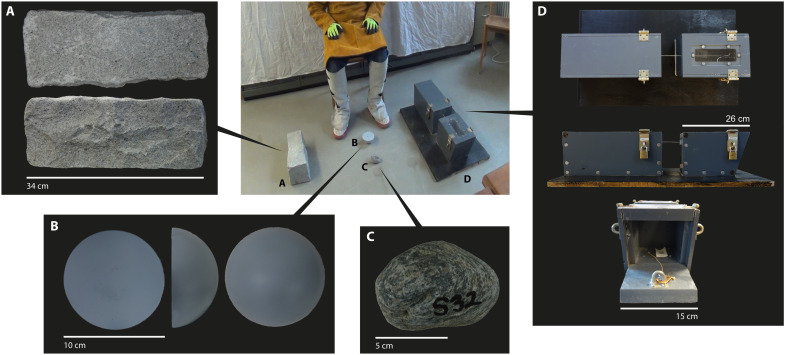
The experimental setup. The experimental apparatus at testing start [here, for participant 3 (P3); seated in the chair at the center, top], including (**A**) the granite block, (**B**) the painted glass hemisphere, (**C**) the river cobble, and (**D**) the puzzle box. This is the view as seen from camera 2 (fig. S1 and Supplementary Text). Photo credits: William D. Snyder and Jonathan S. Reeves, University of Tübingen.

**Fig. 3.**
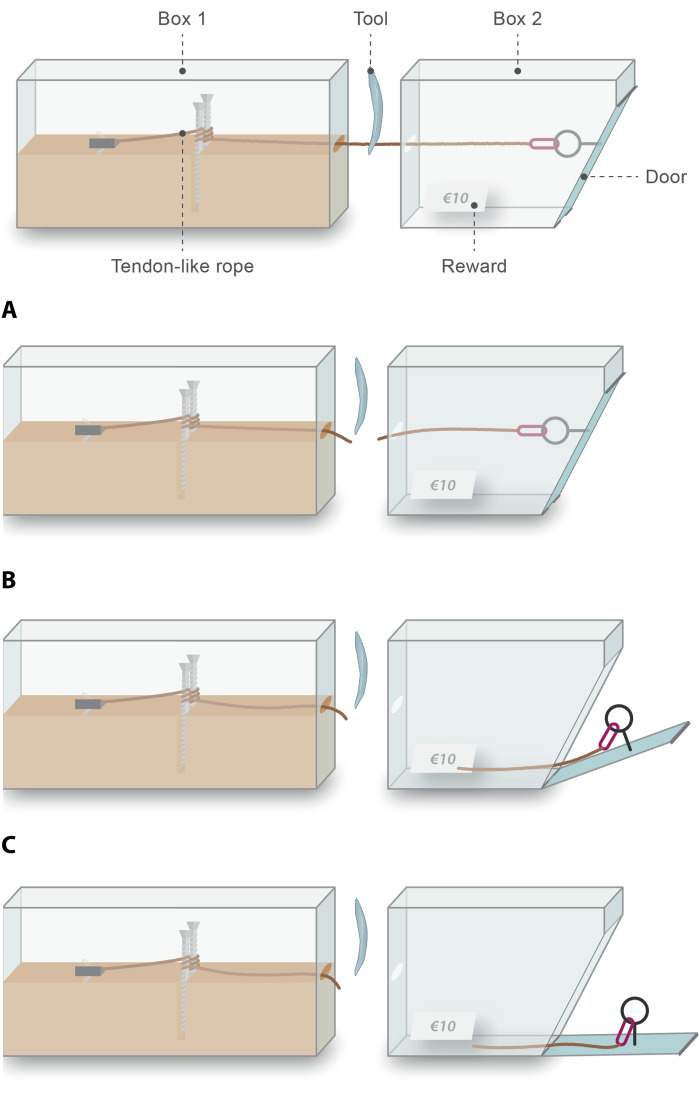
Mechanism for opening the puzzle box apparatus. The apparatus consists of two main compartments (i.e., boxes). A tendon-like rope is led from box 1 into box 2 and then used to fasten shut the door at the front of box 2. Hence, to open the door and access the hidden reward (which is visible to the participant via a window on the top of box 2; fig. S1), the participant must somehow break or sever the rope. In this example, a flake (here, not to scale) was produced from the glass hemisphere and used to cut the rope (**A**), thus relieving the tension in the rope and opening the door of box 2 (**B**). With the door now open, the participant can reach in and remove the reward slip (**C**). In other cases, participants used the core as a cutting tool or opened the door of the puzzle box using a noncutting solution (fig. S2 and Supplementary Text).

Here, we present the results of an island test for early knapping techniques, run using human participants (*N* = 28; *n*_male_ = 14, *n*_female_ = 14). As in earlier nonhuman tests (*32*, *39*–*41*), our human participants were given motivation to make and use cutting tools (a puzzle box containing a reward accessible by severing a rope visibly holding a door closure shut) and access to the necessary raw materials for toolmaking (also known as know-what and know-where information; Figs. 2 and 3 and fig. S1 to S3) (*5*). Beyond this, participants did not receive cultural information (demonstrated, spoken, written, or otherwise) (*3*, *5*) related to stone tools, stone tool types, or stone tool–related know-how, i.e., knapping techniques (Supplementary Text). We also controlled for participant naivety regarding stone tools and early knapping techniques via a questionnaire but only after the test so that the questionnaire itself did not endanger the naivety status of the participants. Subjects that had previously been exposed to knapping techniques (naivety levels 3 and 4) were regarded as non-naïve to the target know-how (Table 1). In these ways, we generated data from an island test condition for early knapping techniques (Supplementary Text). As it could not be established a priori whether and when participants would develop any knapping technique, we tested each participant in a sufficiently long (4-hour) session. Furthermore, all participants were tested alone in single sessions to exclude cultural transmission between individuals and to serve, in each case, as an independent replication of the results.

**Table 1.** Breakdown of participants by naivety level and behavioral outcomes. Reported values are the number of participants that fulfill both conditions (specified naivety level and specified behavioral outcome). The values reported in parentheses are those participants that also fulfill the criteria of the cell but only did so after they had received the impossible flake (Supplementary Text). P6, P21, and P23 all performed knapping techniques in potential toolmaking events before receiving the impossible flake, but only P6 made and used cutting tools afterward. P21 produced potential cutting tools without using them after receiving the impossible flake. P23 did so as well, but this was identifiable only from the artifactual outcomes and not via the coding of the video-recorded behavioral data.

		**Naivety levels**					**Total**
		**Technique-naïve, *n* = 25**			**Non–technique-naïve, *n* = 3**		
		**0**	**1**	**2**	**3**	**4**	
		**No knowledge** **of stone tools or** **knapping**	**Heard of stone** **tools**	**Seen stone** **tools**	**Seen knapping**	**Hands-on** **knapping** **experience**	
**Behavioral** **outcomes**	Performed at least one knapping technique (potential toolmaking)	*n* = 2	*n* = 1	*n* = 22	*n* = 2	*n* = 1	*n* = 28
	Produced at least one potential cutting tool (confirmed toolmaking)	*n* = 2	*n* = 1	*n* = 19 (*n* = 2)	*n* = 2	*n* = 1	*n* = 25 (*n* = 2)
	Produced and used a cutting tool	*n* = 2	*n* = 1	*n* = 19 (*n* = 1)	*n* = 2	*n* = 1	*n* = 25 (*n* = 1)
**Total**							***N* = 28**

In this way, we were able to determine whether naïve humans could reinnovate early knapping technique(s) in the absence of cultural transmission of their know-how. If naïve modern humans in our test can individually arrive upon any early knapping techniques, ideally, by inducing conchoidal fracture, and especially if they would develop all discussed techniques (passive hammer, bipolar, freehand, and projectile techniques) (*16*, *26*, *42*–*45*), then the hypothesis that early knapping techniques do not necessitate know-how copying would be supported. Simultaneously, the hypotheses that posit a necessity for cultural transmission of the know-how of early knapping techniques would become less parsimonious.

## RESULTS

**Fig. 4.**
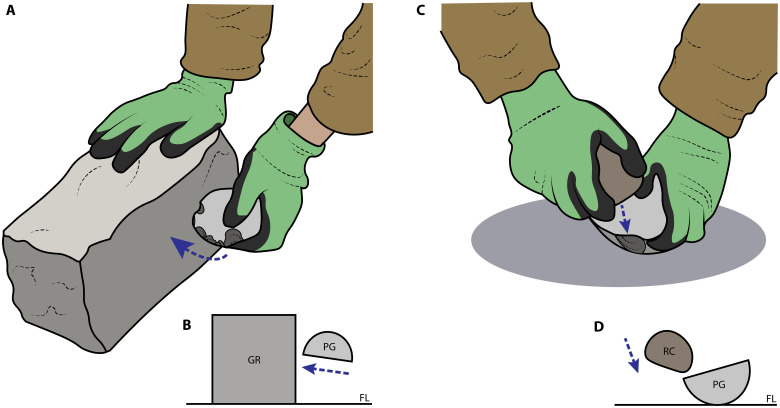
The preferred knapping techniques of the two totally naïve participants (level 0). (**A** and **B**) Passive hammer technique used by P14 (trial 45) shown from the perspective of camera 2 (A) and in profile view (B). (**C** and **D**) Bipolar technique used by P11 (trial 23) shown from the perspective of camera 2 (C) and in profile view (D). Blue, dashed arrows represent the directionality of force of the active element. The abbreviations in the profile views (B and D) stand for the following: GR is the granite block, PG is the painted glass hemisphere, RC is the river cobble, and FL is the concrete floor.

As indicated by the posttest questionnaires, 25 of 28 participants were technique naïve (i.e., had no knowledge of or previous experience with any knapping technique; naivety levels 0 to 2; Table 1 and table S1). Of these 25 technique-naïve participants, 22 participants had only seen stone tools before (naivety level 2; Supplementary Text); one participant had heard of stone tools (level 1); and two participants (participants 11 and 14, henceforth P11 and P14) had been totally naïve concerning stone tools, stone tool types, and knapping techniques (level 0) before testing. Despite the naïve nature of 25 participants regarding knapping techniques, all 25 of these spontaneously used knapping techniques, 22 of whom spontaneously produced and then used cutting tools (two others made potential cutting tools, and one of those two used cutting tools after having been given the impossible flake, i.e., nonspontaneously; table S2 and Supplementary Text). Both totally naïve (level 0) participants individually developed early knapping techniques (Fig. 4) and used the resulting artifacts as cutting tools (Fig. 3 and figs. S2 and S3).

**Fig. 5.**
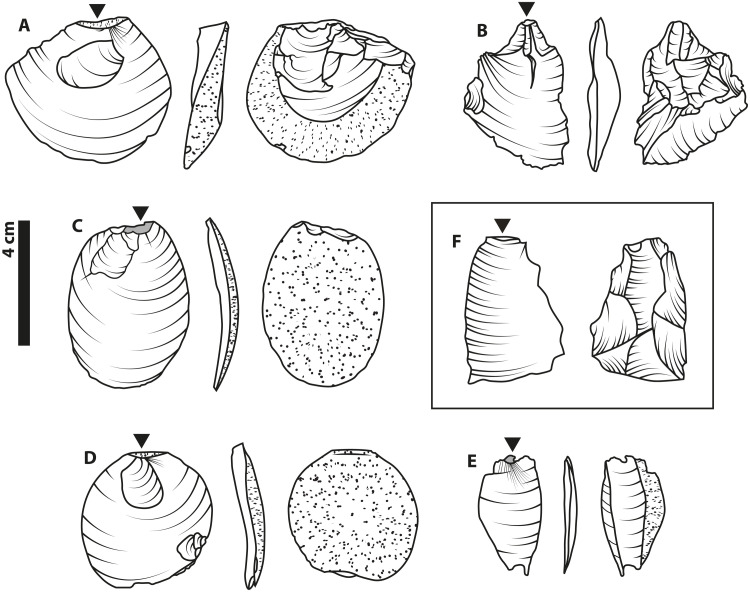
Technological illustrations of flakes. All experimental flakes depicted here were made by individuals who had never before seen the process of stone toolmaking and were used successfully as cutting tools. Experimental artifacts are shown here in ventral, lateral, and dorsal perspective. Flakes are shown in platform-up orientation, with black triangles pointed at the approximate location of the point of percussion. Light gray areas indicate crushing events, while dotted areas indicate cortex (the original painted surface of the glass hemispheres). Participant ID (e.g., P11), participant naivety level (e.g., L0 for totally naive), and the knapping technique that was used to produce the flake are as follows: P11, L0, and bipolar (**A**); P14, L0, and passive hammer (**B**); P7, L1, and passive hammer (**C**); P8, L2, and freehand (**D**); and P15, L2, and projectile (**E**). A premodern hominin-produced flake (**F**) from the site of Gona, Ethiopia (2.6 to 2.5 Ma) (*73*) serves as just one example of a vast diversity of flakes known from the Oldowan and is shown in the box, center-right.

Overall, we recorded 1580 potential toolmaking events (i.e., where the actions therein could have or did lead to the fracture of an object; tables S4 to S8). Of these potential toolmaking events, 1095 (69.3%) resulted in the fracture of an object and subsequent creation of potentially usable cutting edge (confirmed toolmaking events; Fig. 4, fig. S4, and tables S4 to S6 and S9). Note that, as what typically occurs during knapping, a single bout sometimes produced more than one detached piece. The combined artifact assemblage at the end of this study consisted of 1599 objects, with flakes accounting for 73.3% of this total (1172 flakes produced from 33 glass cores; Fig. 5 and table S10).

**Fig. 6.**
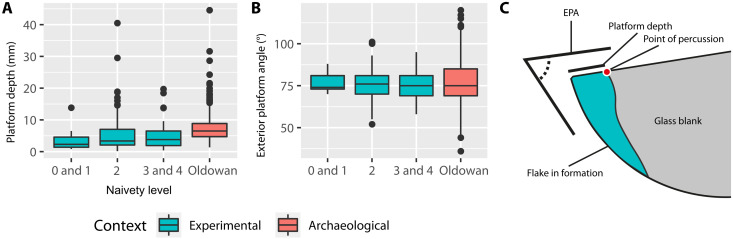
Metric comparison between experimental (*n* = 266) and archaeological flakes (*n* = 620). (**A**) Depth of platforms on experimental (left) and archaeological (Oldowan, right) flakes. (**B**) Exterior platform angle (EPA) on experimental (left) and archaeological (Oldowan, left) flakes. (**C**) Schematic showing the formation of a flake by percussive knapping and the relationship to the aforementioned metrics.

All confirmed toolmaking events for the first totally naïve participant P11 (*n* = 36, 100.0%) involved the application of bipolar technique. The preferred approach (used in more than half of the toolmaking bouts; Fig. 4, C to D; fig. S4; and movie S1) for P11 was to place the core (glass hemisphere) directly on the concrete floor, stabilize the core with one hand, and then strike it from above with the provided river cobble (as a hammer) using the other hand. When considering potential toolmaking events more broadly, P11 also showed evidence for both passive hammer (*n* = 1) and freehand (*n* = 1) techniques. As for products, P11’s toolmaking primarily resulted in the production of flakes (*n* = 35 of *N* = 41 artifacts in P11’s assemblage; Figs. 5 and 6).

The first technique used by the other totally naïve participant P14 was freehand (*n* = 1, 1.9%), although nearly all confirmed toolmaking events for P14 (*n* = 53, 98.1%) involved a variant of passive hammer technique (Fig. 4, A to B; fig. S4; and movie S1), in which the outer edge of the core (glass hemisphere) was struck against the side of the granite block. Regarding potential toolmaking events, P14 additionally engaged in bipolar technique (*n* = 1). Just as with P11, P14’s toolmaking actions produced mostly flakes (*n* = 61 of *N* = 77 artifacts; Figs. 5 and 6).

Among all 28 participants, 22 used more than one technique to successfully create sharp edges/cutting tools. All but one participant demonstrated a preference for one technique over other techniques (Supplementary Text). The total number of techniques expressed by individuals did not differ across naivety levels (χ^2^ = 5.7528, df = 4, *P* = 0.2184, Kruskal-Wallis test).

The three early knapping techniques (passive hammer, bipolar, and freehand) reinnovated by the two totally naïve participants were also often used by other participants. The fourth coded category, projectile technique, although not shown by either totally naïve participant, was developed by several other participants, all of whom were knapping technique naïve (*n* = 1 for level 1 and *n* = 9 for level 2; Fig. 4, fig. S4, movie S1, and Supplementary Text).

Across all attempts and across all participants, bipolar technique was the most frequent category of potential toolmaking (574 events, 36.3%), followed by passive hammer (559 events, 35.4%), freehand (410 events, 25.9%), and projectile (37, 2.3%). In terms of individual preference for technique, the preferred technique in potential toolmaking events was most often bipolar technique (*n* = 10, 35.7%), followed by passive hammer (*n* = 7, 25.0%), anvil-oriented (preference for subsequent or alternating use of passive hammer and bipolar technique, *n* = 5, 17.9%) freehand (*n* = 4, 14.3%), and opportunistic (no technique preference, *n* = 2, 7.1%; tables S6 and S7). As far as confirmed toolmaking events, bipolar technique was the most frequent (466 events, 42.6%), followed by passive hammer (398, 36.3%), freehand (226, 20.6%), and projectile (5, 0.5%; table S9). Individualized preferences for techniques were distributed between bipolar (*n* = 13, 48.1%), passive hammer (*n* = 10, 37.0%), freehand (*n* = 3, 11.1%), and opportunistic (no technique preference, *n* = 1, 3.7%; table S6).

**Fig. 7.**
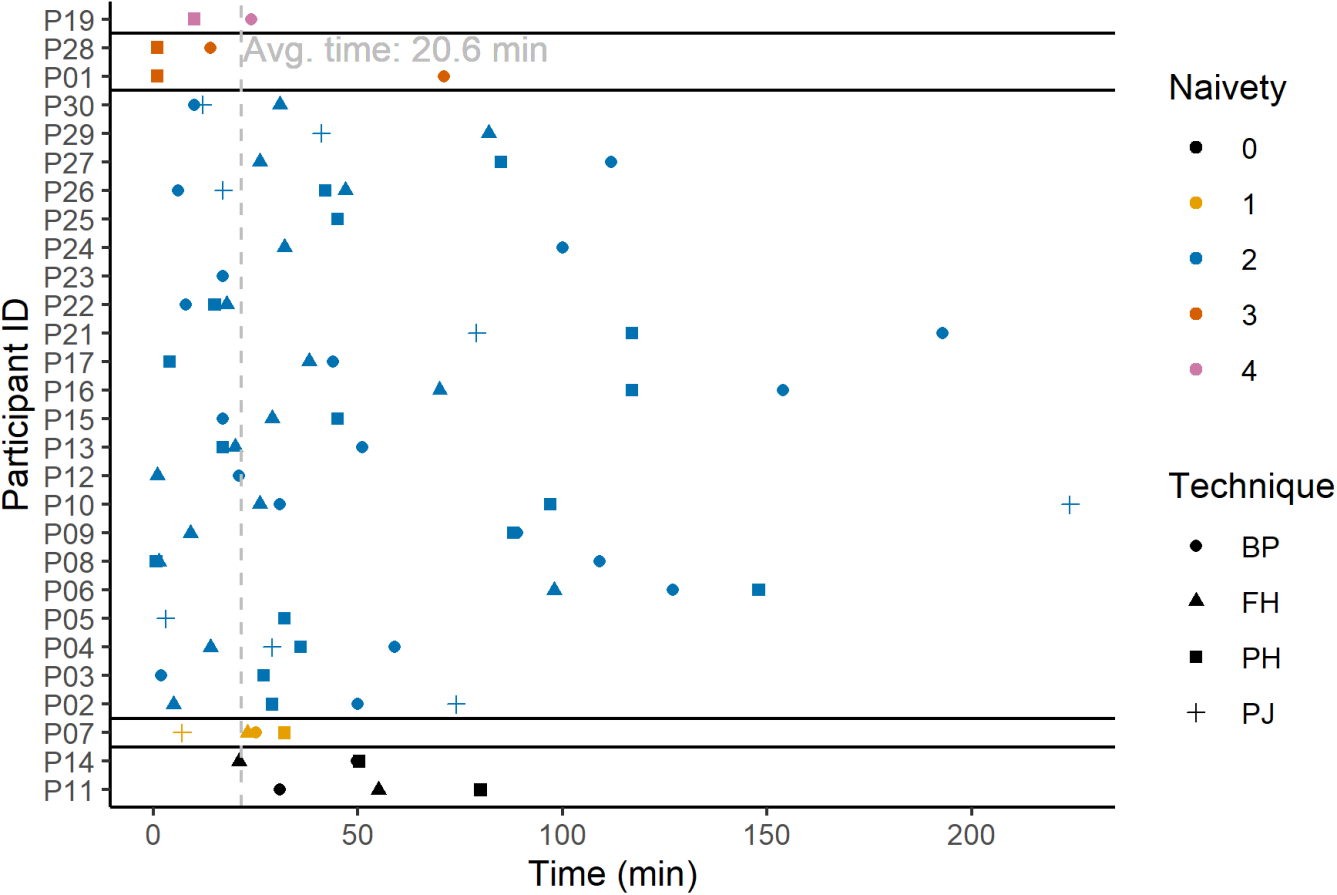
Timeline of first toolmaking innovations by technique. The timing of each innovation of a potential toolmaking technique for each participant (testing time elapsed at the start of the bout, in minutes). Shape of data points corresponds to the technique used in the behavioral bout, and color of the data points corresponds to the naivety of the participant based on the posttest questionnaire (see legend). The gray, dashed, vertical line represents the average timing of the first innovation of any technique by each individual.

The first instance of potential toolmaking generally occurred within the first hour of testing (*t ®* = 20 min and 38 s, SD = 25 min and 13 s; Fig. 7 and Supplementary Text), with the confirmed first toolmaking also being relatively expedient (*t ®* = 35 min and 58 s, SD = 56 min and 20 s; Supplementary Text). Participants typically engaged in numerous noncutting solution attempts (e.g., simple percussion with the cobble against the rope, rubbing a blunt object against the rope, and untwining the fibers of the rope with their fingers) before they showed any potential toolmaking behaviors (mean = 11.7 attempts, SD = 16.7 attempts). This count of attempted noncutting solutions to the puzzle box increases when only considering the first confirmed toolmaking event (mean = 17.9 attempts, SD = 26.1 attempts). Only three individuals (P1, P3, and P28) produced and used a cutting tool as their very first solution to the puzzle box during the test. Some participants attempted noncutting solutions even after at least one initial success in making and using a cutting tool (most notably, P2; Supplementary Text). One of the two totally naïve participants, P11, produced their first cutting tool after 20 min and 52 s of testing time. The second of the two totally naïve participants, P14, produced their first cutting tool after 23 min and 23 s of testing time. There was no significant difference between naivety categories in terms of the timing of the first innovated potential toolmaking (χ*^2^* = 5.8528, df = 4, *P* = 0.2104, Kruskal-Wallis test) or of the first confirmed toolmaking (χ*^2^* = 5.3537, df = 4, *P* = 0.2529, Kruskal-Wallis test).

Participants across naivety categories did not differ in terms of the number of flakes produced (χ*^2^* = 3.0340, df = 4, *P* = 0.5522, Kruskal-Wallis test). Experimental flakes had statistically shallower platforms [platform depth (PD)] than Oldowan flakes (χ*^2^* = 111.96, df = 3, *P* = 2.2 × 10^–16^, Kruskal-Wallis test; Fig. 6A, table S11, data file S1, and Supplementary Text). All exterior platform angles (EPAs) measured on experimental flakes (range of 52° to 101°) fell within the expected range for Oldowan flakes (Fig. 6B, data file S1, and Supplementary Text) (*46*). EPA was not significantly different between naivety groups and between naivety groups and Oldowan assemblages (χ*^2^* = 0.53688, df = 3, *P* = 0.9107, Kruskal-Wallis test; table S12).

**Fig. 8.**
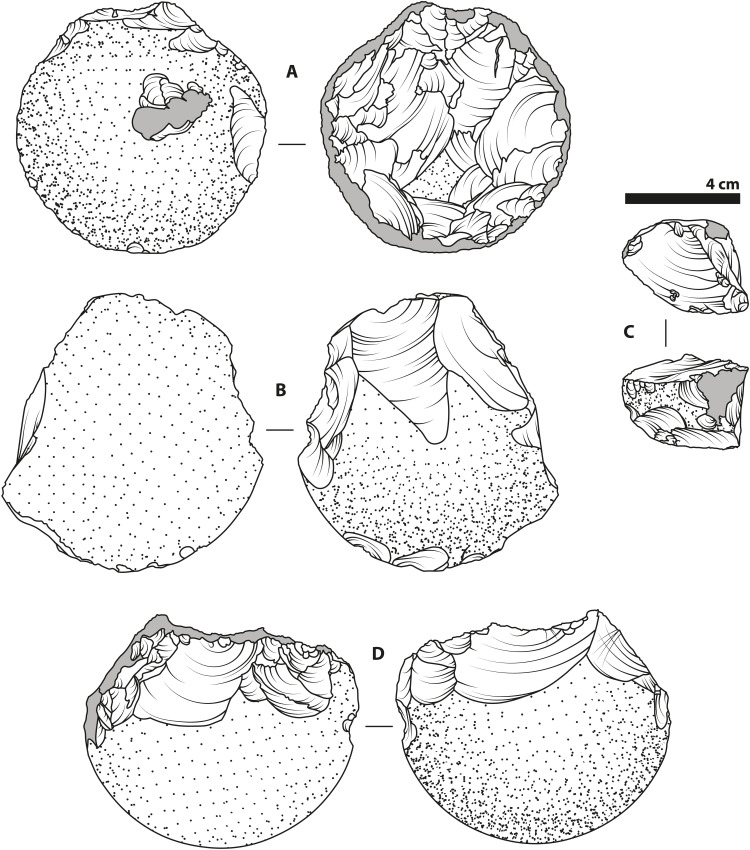
Representative cores from this study. Illustrations based on a selection of cores produced by the participants in this study. As with the Oldowan artifacts (Fig. 1), represented here are a variety of core types including a radial core produced by P14 (**A**), a unifacial core or “chopper” produced by P7 (**B**), a multifacial core produced by P17 (**C**), and a bifacial core produced by P19 (**D**). Light gray areas indicate areas with signs of crushing, battering, and incipient step fractures, while dotted areas indicate cortex (the original painted surface of the glass hemispheres).

Patterns of removals (Fig. 6C) on the cores themselves varied across individuals (Fig. 8). The most frequently appearing morphology were multifacial cores (*n* = 9; Fig. 8C), which were heavily reduced without clear evidence of systematic exploitation of specific knapping platforms. After this, the core type with the most frequent and identifiable pattern of removals were radial cores (*n* = 8; Fig. 8A, fig. S5, and movie S2). These cores involved the exploitation of the rounded surface of the hemisphere as a striking platform, causing mostly removals from the flat face that are directed at the midpoint of the circle. Knapping of a single exploitation surface or on two adjacent exploitation surfaces on one edge of the blank produced cores similar to unifacial (*n* = 5; Fig. 8B) and bifacial (*n* = 2; Fig. 8D) choppers, as well as heavy-duty (*n* = 2) scrapers. One Karari-style scraper (*n* = 1) (*47*) was also produced by the complete exploitation and removal of the rounded surface of the blank.

## DISCUSSION

The origin of cultural transmission of know-how in the human lineage is a central focus of anthropological research. Previous claims based on correlational data from experiments in which humans learned to knap after cultural access to stone tools and/or knapping techniques align this origin with the earliest flake and core technologies (*10*, *22*, *23*). Here, we tested for the spontaneous reinnovation of early knapping techniques in the absence of opportunities for cultural transmission of their know-how. We validated our approach with posttest questionnaires, which established the knapping technique-naïve nature of the majority (*n* = 25) of our participants. We provided them only with motivation (a baited puzzle box with a rope-lock) and the necessary raw materials for knapping (know-what and know-where) (*3*, *5*). Despite most participants’ naivety to knapping know-how at the start of and the absence of know-how transmission during our experiment, all our technique-naïve participants proved capable of individually reinnovating early knapping techniques. Two participants were found to be totally naïve to the very concept of stone tools, yet even these participants reinnovated knapping and the usage of cutting tools. This finding calls for a reinterpretation of the conclusions from previous knapping studies regarding modern human knappers and regarding premodern hominin knappers, seeing as these earlier studies did not truly test for technique-naïve individual performances. Early knapping techniques, here, did not necessitate cultural transmission of their know-how.

Contrary to claims that early knapping techniques must be culturally transmitted (*7*–*10*), we found that all four of these techniques can be individually developed in the absence of such cultural transmission of their know-how. The vast majority of our (mostly technique-naive) participants reinnovated early knapping techniques, and this included the totally naïve participants we tested, who had not only no previous cultural access to knapping techniques but also no previous knowledge of stone tools and stone tool types. Overall, we detected reinnovation of all four of the discussed early knapping techniques by technique-naïve participants (passive hammer, bipolar, freehand, and projectile) (*16*, *26*, *42*–*45*). Even the two technique-naïve participants who were also naïve to stone tool concepts innovated three of the four knapping techniques (only projectile technique failed to appear in these two participants; Fig. 4, fig. S4, and movie S1).

In previous knapping studies, there was a restricted expression of techniques, as participants’ behaviors were often socially guided (and thus, limited), especially by prescribing them, via demonstrations or restrictions in the experimental setup (e.g., lack of potential anvils), specific toolmaking techniques that the participants were expected to express. Unless otherwise required by the testing procedure (i.e., for the study of passive hammer or bipolar techniques) (*26*, *48*), freehand technique was often the default, expected means of knapping in earlier experimental studies despite existing evidence of other techniques throughout the record (*26*, *42*, *43*, *45*). Note that even where demonstrations exclusively used freehand technique, there are still documented cases of apparent reinnovation of bipolar technique (*24*, *49*, *50*). Overall, earlier studies seem to have triggered higher frequencies of expected techniques in participants via social means and/or the physical setup of the task at the expense of alternative techniques that were nevertheless in full reach of participants’ individual skill development. In our own study, with neither social nor physical guidance regarding knapping know-how (or restrictions on the expression of this know-how), participants were free to express, and promptly expressed, a wider range of techniques. In addition, there were many individuals in our study that switched between techniques, but these were likely a response to the dynamically changing form of the core (e.g., switching from freehand or passive hammer to bipolar when cores are more reduced and have fewer workable angles) (*26*, *51*). With regard to the subsequent tool use, it is worth pointing out that our protocol theoretically enabled, and to some degree, possibly even channeled the participants toward, a perceived need to use cutting tools. Nonetheless, our setup empirically proved to be sufficiently opaque, as most participants did not immediately produce knapping techniques or cutting attempts. Generally, the participants first attempted numerous noncutting solutions to the task, and some even pursued these after having already successfully used a cutting tool (Supplementary Text). Such channeling toward a need for cutting, even if it occurred, still would not have led to know-how transmission regarding specific knapping techniques.

All technique-naïve participants (*n* = 25) reinnovated knapping techniques, and among them, we found 20 cases of reinnovation of passive hammer technique, 22 cases for bipolar technique, 20 cases for freehand technique, and 10 cases for projectile technique (reinnovation here meaning the first use of a particular early knapping technique by a single individual; Fig. 7 and Supplementary Text). Reinnovation by a single naïve subject, of any species, would logically suffice to show that any affected technique does not require cultural transmission. Therefore, with our many independent cases (i.e., in-study replications) of reinnovation (here, in humans), we provide ample empirical evidence that it is possible, in principle, that all four of the main proposed early knapping techniques (*16*, *26*, *42*–*45*) can be individually reinnovated.

Parallel theoretical and empirical approaches have already worked under the alternative assumption that these knapping techniques, their know-how, could have likewise been individually derived in the case of premodern hominins (*34*, *35*). The fact that another species, here, modern humans (although again, similar findings from other species would have fulfilled the same logic), can individually reinnovate all of these techniques provides empirical realism for the working hypothesis of such individual know-how reinnovation in other species, particularly by premodern hominins, but in addition, humans are the phylogenetically closest relatives of premodern hominins, meaning that the results of human tests hold particular relevance to the larger context of technological evolution. Further support is found in knapping technique reinnovation by another phylogenetically close primate species (unenculturated, untrained, and technique-naïve orangutans) (*41*), although only passive hammer technique was spontaneously reinnovated in that study. The spontaneous reinnovation of at least some knapping techniques in unenculturated nonhuman primates additionally shows that there is no logical necessity for even an indirect pathway of cultural transmission to knapping know-how.

With regard to material outcomes, flake production by conchoidal fracture is the hallmark physical component of Oldowan knapping, as well as of later Mode 1 technology (*12*, *14*, *15*), and is also a feature that previous experimental studies have focused upon (*10*, *20*, *21*, *23*, *40*). The identification of a large portion of the artifacts produced in our study as conchoidally produced flakes with EPAs in the same range as Oldowan material provides validity of our data to both the previous inexperienced knapper data under social learning conditions and the Oldowan record (Fig. 6B and data file S1). This reinforces the view that the material-dependent mechanics of knapping help guide individuals’ know-how acquisition over time, as there is only a limited span of possibilities for conchoidal fracture. That knapping by our naïve participants proved sufficient to generate Oldowan-like artifacts (Figs. 5, 6, and 8; fig. S5; movie S2; and Supplementary Text) should perhaps be expected, given the present interpretation that these core forms were the unintentional result of “least effort flaking” (*14*), a view backed by recent empirical work on core form outcomes of purely stochastic knapping (*52*). Our data provide further evidence that mental templates are not a necessary component of Oldowan-type flaking (*14*, *52*), with the affordances of the initial blank probably being most important in determining the patterns of reduction and the final core outcomes (e.g., the preponderance of radial cores from hemispherical blanks) (*14*).

External validity is a pertinent component of building an analogy between present experimental results and the prehistoric record that we seek to understand (*53*, *54*). When designing knapping studies, there is an inherent trade-off (*24*) between experimental control on one hand, which typically improves precision, reliability, and measurability, and naturalistic approaches on the other (i.e., using only those precise rock types and forms available to the hominins), which improve external validity at the expense of control. Here, we opted for high experimental control. We provided participants with hemispherical glass blanks, which were suitable for easy knapping (in terms of material and workable platforms), but we believe that this was a justified choice also in terms of external validity. Regarding the material, glass (albeit volcanic and not artificial glass) was exploited in prehistory, including in Oldowan contexts (*55*), and in any case, glass conchoidally fractures not unlike other raw materials (*56*). As for shape, hemispheres (or split cobbles) are also a known property of the Oldowan (*14*). Our participants produced all the core outcomes that are expected from such hemispherical blanks, based on the Oldowan record (*14*).

Small differences between our experimental flakes and Oldowan flakes were detected. In this instance, the flakes from this study had shallower platforms than flakes from an Oldowan reference dataset (*46*). However, our study falls in line with the outcomes of previous human knapping studies, which have repeatedly shown that flakes made by inexperienced knappers are distinguishable from both flakes found in the archaeological record and those made by human expert knappers (*30*, *31*, *57*). We may therefore surmise that our participants entered a learning curve that is already known from previous human knapping studies, only via an alternative, individual, route. Even in group learning scenarios with ample social learning opportunities (and in 2 hours of testing), individuals in another knapping study did not achieve expert Oldowan knapping skills (*24*).

Given our findings, a more parsimonious explanation for the observed differentially increased performances in prior knapping studies involving know-how transmission opportunities (*10*, *22*–*24*, *33*) is that these opportunities might have merely sped up skill acquisition instead of being strictly necessary. In line with this is also the fact that acquisition of knapping techniques was very swift in these earlier studies (a few minutes sufficed) (*10*). Although, before our study, we hypothesized that at least some naïve participants would reinnovate knapping techniques, the novelty of our approach meant that we were unable to predict how long human participants would require to individually reinnovate any knapping techniques, so we provided participants with an elongated learning time (4 hours). These long learning times proved to be unnecessary, as technique reinnovation usually required only tens of minutes (Fig. 7 and Supplementary Text). Future studies should however test whether technique-naïve humans, as tested in our study, would eventually reach Oldowan-evidenced expert-level performance on their own if simply afforded sufficient time and, with it, opportunities for individual learning. We tentatively hypothesize that such a realization of skill acquisition, interpreted from artifacts at some early Oldowan sites (*30*), can be achieved solely via individual, hands-on knapping practice (*19*, *28*, *58*). Ideally, future studies would investigate this and other hypotheses using a longer-term individual learning trajectory under island test conditions, as well as comparing reinnovation and individual learning of knapping techniques cross-culturally and developmentally (i.e., by studying knapping abilities of children).

Contrary to the interpretation of data derived from earlier experimental studies that provided human learners with various types of cultural access to stone tools and/or their production techniques, cultural transmission of knapping know-how specifically proved unnecessary for the development of early knapping techniques by human participants, and by extension, for the generation of flakes and artifactual assemblages with a general Oldowan-like character. Our data thereby logically contradict claims and assumptions that the existence of early knapping techniques per se necessitated cultural transmission of knapping know-how in the past, as well (*7*–*10*). Our findings logically show that, all, early knapping techniques can principally be individually developed. In light of these latest findings, the interpretation of early knapping techniques and artifacts from the Oldowan warrants further inspection.

Island tests are a powerful, discriminatory tool for determining whether certain transmission mechanisms are necessary for the expression of target behaviors (*5*). Here, we used the island test method specifically to control for the cultural transmission of knapping know-how. The application of this method should, however, not be taken to mean that “natural Island test conditions” would have been frequently, or solely, encountered by premodern hominins. Such conditions could have sometimes existed (*34*), but many populations of premodern hominins would not have encountered them. However, even where premodern hominins would have grown up in situations in which others in their social group already knapped, this would not have meant that the know-how of knapping techniques was culturally transmitted. Instead, we advocate for serious consideration of a recently introduced alternative hypothesis, namely, a minimal culture model, of Oldowan technology.

Within a minimal culture model of the Oldowan, as is proposed for nonhuman primate cultures (*36*, *37*), social learning very likely existed but did not transmit knapping know-how. Instead, other types of social learning, likely present at the time, would have merely affected the frequencies and speed of serial, but still individual, reinnovations of this know-how within and across populations. The resulting minimal culture would then have been tied to socially affected frequencies of know-how reinnovation but not to cultural transmission of know-how. For example, in living groups of premodern hominins, these social influences on know-how reinnovation would have resulted from the transmission of other types of information such as know-what (e.g., knappable stone types) and know-where (e.g., whereabouts of carcasses) information. The transmission of these other types of information would then have secondarily resulted in serial, but ultimately still individual, reinnovations of knapping know-how, a process more akin to catalyzation than to copying (*3*, *5*, *37*). Once (re)innovated, these techniques would then not only have persisted in their populations but would have been more readily and more often reapplied over each individual innovator’s lifetime, overall leading to increasing skill levels on both the individual and the group level. In this scenario, proximity to and engagement with the socially provided context (including locations, targets, and types of requisite materials) would therefore have sufficed to produce group-level phenomena, minimal cultures, without any theoretical requirement for the specific know-how itself to be transmitted. This account therefore stands alone in not requiring the assumption of the presence of know-how copying skills during the Oldowan. To underline this point further, the minimal culture model, a cultural model, after all, still accounts for general processes of cultural transmission that generate population-level patterns (which did exist during the Oldowan) (*11*, *45*), while it parsimoniously does not require the specific assumption that cultural transmission of know-how was present, a skill often considered key for the cumulative cultural evolution of know-how seen in modern humans (*1*–*4*).

Within the minimal culture model, the, likely multiple independent (*13*, *45*, *59*), origins of the Oldowan (and Mode 1 technology more broadly) and the succession of later technological traditions would thus be better explained in terms of ecological and biological affordances, including mechanical affordances of raw materials (*60*). In such a scenario, instead of cultural transmission of know-how, other cognitive mechanisms (e.g., working memory, spatial and causal reasoning, and motor coordination) (*20*, *28*, *35*, *58*, *61*–*66*) would have played a proportionally larger role in the (slow) evolution of early hominin technologies. Given the absence of spontaneous production or use of flakes via knapping in chimpanzees (*40*), developments in some of these or related cognitive traits after the divergence of the human and Pan lineages could more cogently explain the emergence of the Oldowan and perhaps subsequent industries (*34*, *35*), although at least one knapping technique (passive hammer) seems to be also within the spontaneous individual innovative realm of a more distantly related ape species, orangutans (*41*).

On the grounds of the results presented here, early core and flake technology can no longer be used as unequivocal evidence for an early onset of cultural transmission of know-how, suggesting a reexamination of such beginnings of the cumulative cultural evolution of know-how. Absent new lines of evidence, the earliest unequivocal evidence for technique transmission, and with it, cumulative culture of know-how, should be pushed forward to a later time, the identification of which should be the focus of future experimental research.

## MATERIALS AND METHODS

### Experimental design

Thirty participants were recruited via online and newspaper classified listings in a town in southwestern Germany. Two participants were tested, but their data were excluded (because of safety concerns and experimenter error, respectively). A final sample of 28 tested participants (14 female and 14 male) are reported here. Participants in this study were “WEIRD” (referring to people from societies that are “Western, Educated, Industrialized, Rich, and Democratic”) (*67*). This choice provides commensurability with previous knapping studies, which likewise used participants from WEIRD populations. WEIRD individuals are nonetheless appropriate for this type of study (i.e., an island test) since they are generally unlikely to be exposed to knapping experiences in their daily lives.

At the start of testing, each participant was provided with one spray-painted glass hemisphere (10 cm in diameter and 4 cm in height; note that hemispherical blank forms or “split cobbles” are known from the Oldowan) (*14*), one locally sourced river cobble, and a large rectangular granite block (Fig. 2, fig. S1, and table S3). Participants could receive replacements for the hemispheres and cobbles if the materials were deemed exhausted according to the participant’s own evaluation. The puzzle box afforded the use of tools and consisted of a box with an enclosed reward accessible by severing a rope, a “tendon” box (Figs. 2 and 3 and figs. S1 to S3) (*40*, *41*). Because of the length of testing, both participant and experimenter(s) were given chairs. All tests were recorded using three separate digital camcorders, mounted on tripods at designated spots around the testing area. Participants were remunerated with €12 per hour plus any attained reward money.

Participants were not given any relevant information (verbal, written, or visual) about knapping or stone artifacts before or during the experiment. The participants were merely instructed to procure the reward from the puzzle box using the available materials in the testing space. Participants were tested individually to further prevent any transmission of information and to ensure independent replication. The experimenter abided by a predetermined script to avoid phrasing that might reveal the goals of the experiment (e.g., the experimenter referred to the cores, flakes, and other materials exclusively as “objects” and never specifically mentioned “cutting”; Supplementary Text). Overall, this setup allowed us to test whether participants would produce and use glass or stone cutting tools by any variant of knapping technique, without compromising their naivety. This also ensured that the spontaneity of their technique innovations could be verified.

Participation was contingent upon informed consent and fulfilment of eligibility criteria (ethical approval by the Ethics Committee for Psychological Research, University of Tübingen, confirmed on 12 June 2019). Prestudy information for the participants only indicated that this would be a study on “human problem-solving abilities” and did not mention stone tools, their form or manufacture techniques. This deception was necessary because transparency about the study’s aims would have compromised the participants’ naivety.

Because of health and safety regulations, participants and experimenter(s) wore safety gear and were not permitted to remove any of it while in the testing area. Each participant was tested for a maximum of 4 hours (two tests ended early by participant request). Just before testing, participants were given explanations of the testing setup, including safety precautions and general study rules (e.g., no access to smartphones and no questions about specific solutions). The experimenter also clarified the mechanics of the puzzle box (i.e., that a visible rope prevented a door from being opened, thus further blocking access to the reward inside). After the initial study explanation, the experimenter signaled to start, at which point, the participant could begin pursuing solutions. If participants used a solution other than creating and using a cutting tool, then the solution was noted and named live by the experimenter, and the participant was then instructed not to repeat this solution and to instead pursue other (unnamed) avenues of opening the puzzle box. The puzzle box was then reset and rebaited. The puzzle box was also reset and rebaited upon successful creation and usage of a cutting tool (either as a detached piece or cutting edge on a core).

Used detached pieces were retrieved by the experimenter and bagged and labeled, while successfully used edges on a core were visibly marked by the experimenter (with a pen) and verbally designated as unusable. This procedure simulated edge wear (as occurs in naturalistic conditions) and thus encouraged the production of new edges. If the participant did not produce a cutting tool after 2 hours, they were provided an “impossible flake” (i.e., a tool with a functional shape, a wedge-like triangular prism made of glass and spray-painted, but impossible to recreate via knapping) for exactly one trial. Puzzle boxes were baited with paper slips designating monetary rewards (Figs. 2 and 3 and figs. S1 to S3). Participants received this monetary reward (as credit) for each time that they opened the puzzle box, regardless of the solution type.

After approximately 5 min of fruitless toolmaking attempts and/or total inaction (at any point during testing), participants were asked whether they would like to receive a new blank. If participants requested a new blank (i.e., deemed exhaustion of the previous one), then the old core and debitage were all collected and bagged with labels. Otherwise, collection, bagging, and labeling of cores and debitage occurred at the end of the test.

To ensure that participants were naïve and remained naïve before and during testing, it was necessary to collect data on the participants’ previous experience with stone tools after the test. Thus, once the testing time was complete, participants filled out a questionnaire about their prestudy experiences, of any type, with stone tools.

Behaviors were coded live by the experimenter using a paper coding sheet. After the test ended, video recordings were processed (angle selection and anonymization) and then coded. First, behavioral bouts (actions or action sequences with a definable start and end point) were identified as involving toolmaking, tool use, or simultaneous toolmaking and tool use. We coded events as potential toolmaking, both when actions did lead or could have led to fracture of an object and, with it, the creation of a potential cutting tool. Confirmed toolmaking events, on the other hand, were those where the fracture of an object could be determined visually from the video recordings (fig. S4). The dichotomy of potential versus confirmed toolmaking was implemented as the intentions of the participants are not strictly known (unlike other studies, where the intentions are prescribed by the experimenters and therefore reasonably identifiable) (*10*, *11*, *22*–*24*). Toolmaking bouts were further assigned to one of four early knapping technique categories: freehand, passive hammer, bipolar, and projectile. Freehand, passive hammer, and bipolar techniques are all specifically associated with Oldowan technology (*42*, *43*), while passive hammer (*44*) and bipolar technique (*16*, *26*) are also associated with purported and hypothetical pre-Oldowan technologies. Projectile technique is not typically associated with the Oldowan proper but has been cited as a potential pre-Oldowan behavior due to its expression by the captive bonobo Kanzi and may have played a role in the innovation of knapping by naïve Oldowan hominins (*26*, *39*). We used a “constellation-based” approach for the assignment of toolmaking bouts to one of the technique categories; this method was based on earlier approaches (*68*) but modified further so that technique classification was fully neutral in terms of intention, outcome, and mechanic (e.g., percussion, friction, and pressure). In this approach, objects are identified using neutral terms such as active, passive, auxiliary, and target element, instead of already interpretative archaeological terms (e.g., “hammer” or “anvil”). If a participant used a technique in ≥50% of toolmaking events, then this was defined as a preference.

For interobserver reliability calculation, a subset of data (*n* = 7 participants; 25.0% of participants) was coded by a hypothesis-unaware individual (J. Keppeler) according to the above criteria. The coding from the experimenter and the reliability coder were then compared.

Participants’ posttest questionnaire answers were used to rank the naivety of the participants, with a minimum rank of 0 for totally naïve individuals (with no theoretical or practical knowledge of stone tools) and a maximum rank of 4 for individuals with at least some previous hands-on experience with stone tools and knapping.

All detached pieces used by the participants as tools were immediately collected, labeled, and bagged for later analysis. Cores and all associated debitage (no matter how small) were collected and labeled either at the end of the test or if the core was exhausted. Used tools of any size and debitage elements equal to or above 2 cm in maximum dimension (*69*) were subject to categorization as flakes or angular fragments (*13*) and a full attribute analysis, two attributes of which are reported here. PD was measured using digital calipers. For EPA, the analyst made three separate measurements of the angle with a goniometer and then averaged the three measurements (all reported EPAs are thus averaged values). Values for PD and EPA from the experimental flakes were compared with a reference dataset (*46*) from Oldowan sites (for our purposes, flakes that were labeled in this dataset as “Oldowan,” “Developed Oldowan,” and “Karari” were combined into one Oldowan technological category). For the cores, basic technological analysis was performed to elucidate the reduction strategies implemented by the participants (*70*) and the basic typology of the final cores (*14*, *71*).

### Statistical analysis

Statistical analyses and graph building were performed using RStudio (*72*). Because of the non-normality of the data and the small size of the sample for the different naivety categories, nonparametric tests [Kruskal-Wallis test for one-way analysis of variance (ANOVA) and Wilcoxon rank sum tests for pairwise comparisons] were used. Tests were two sided (per the default setting in RStudio).

Cohen’s kappa tests for interobserver reliability were used to measure agreement between the coding data of the experimenter (W.D.S.) with the coding data of the naïve secondary observer (J. Keppeler). Cohen’s kappa tests were used on count data (frequency of a behavior or behavioral character) and Boolean data (presence/absence of a behavior or behavioral character). Similarly, a Cohen’s kappa test was performed for interobserver reliability of flake identification, involving a comparison between the typing data from the experimenter (W.D.S.) and the hypothesis-naïve secondary observer (A. Falcucci).

## Supplementary Materials

### This PDF file includes:

Supplementary Text Figs. S1 to S5 Tables S1 to S12 References

## Notes

View/request a protocol for this paper from *Bio-protocol*.
