# Supplementary material for: Early knapping techniques do not necessitate cultural transmission
Source: Sci Adv. 2022 Jul 6;8(27):eabo2894. doi: 10.1126/sciadv.abo2894 (PMC9258951; doi:10.1126/sciadv.abo2894)
Supplement: Supplementary file 1 — Supplementary Text Figs. S1 to S5 Tables S1 to S12 References [file sciadv.abo2894_sm.pdf]

Science Advances
AAAS

Supplementary Materials for

Early knapping techniques do not necessitate cultural transmission

William D. Snyder *et al.*

Corresponding author: William D. Snyder, wdspaleo@gmail.com; Claudio Tennie, claudio.tennie@uni-tuebingen.de

Sci. Adv. **8**, eabo2894 (2022)
DOI: 10.1126/sciadv.abo2894

The PDF file includes:

Supplementary Text
Figs. S1 to S5
Tables S1 to S12
Legends for movies S1 and S2
Legend for data S1
References

Other Supplementary Material for this manuscript includes the following:

Movies S1 and S2
Data S1

Supplementary Text

Glossary

Cumulative culture of know-how may refer to process (the process of cultural cumulation) and products (e.g., culturally evolved behavior patterns such as human dances, words, and material artefacts). With regards to the process, modern human culture cumulates know-how, by enabling the transmission of know-how via special social learning mechanisms (namely those capable of transmitting know-how). Previously transmitted know-how can then act as the steppingstone for additional know-how innovations, and where the latter is therefore culturally derived (and dependent on the formers' presence). Cumulative culture creates cultural lineages of know-how and it creates know-how that exceeds the abilities of single individuals to re-innovate independently (*1-4, 38, 75, 76*).

Know-how is information residing in brains regarding the specific *form* of behavior and/or artefacts (e.g., action sequences and technique in the case of knapping) (*3*); a somewhat overlapping definition is otherwise sometimes referred to as procedural knowledge (*77, 78*).

An industry refers to the broad association of archaeological sites with a shared basic technology, consisting of knapping techniques, reduction strategies, and artefact typologies (i.e., forms).

Knapping is the act of removing sharp pieces - flakes – from suitable stone or other materials via conchoidal fracture (*15*). Generally, removals are initiated via forces like percussion or pressure on a knapping platform (i.e., a surface on a core with suitable angles and area) (*12, 14, 15*).

Conchoidal fracture is the specific physical process by which raw materials for prehistoric stone tools were typically generated (*15*). Conchoidal fracture results in the creation of specific attributes on both cores and flakes that can help identify the specific techniques and procedures

that were used to produce stone tools. Conchoidal fracture leads to sharp edges – likely a main goal of their producers.

Flakes are chips of stone or other raw materials that are removed from a suitable core via knapping (*12, 15*).

Cores are objects made of suitable stone or other raw materials from which removals are made (*12, 14, 15*), therefore showing the negatives of flake removal/reduction processes. In contrast, blanks refer to the initial, unmodified state (i.e., with one removal, a blank essentially becomes a core).

<u>Raw materials</u>

Clear, uncolored glass hemisphere paperweights were purchased from online wholesalers. The hemisphere was standardized in terms of shape and mass (diameter: 10 cm, height: 4cm, mass: 600g). The glass blanks were spray-painted with light grey spray-paint to create an artificial 'cortex' for purposes of concealing the material properties from the test participants and for improvement of attribute measurements post-test.

We note that many participants (n = 13) also made sharp stone tools from non-glass materials, i.e., from the river cobbles and the granite block. Some continued to attempt removals from the river cobbles even after they had already successfully produced glass cutting tools.

Overall, thirty-five glass blanks were provisioned to participants.

Participants were provided a river cobble at the start of the test. River cobbles were purchased from a local construction supplies firm (Natursteinpark Tübingen). These were then selected for use as hammerstones (at least, as predicted by the experimenters) in accordance with the means of masses reported for Oldowan sites at Olduvai Gorge Beds 1 and 2 (Table S3) (*68*), since the data were published and already used to design hammers used for knapping experiments with great apes (*39*). Standard deviations were compared as well to make sure that the experimental selection was similar in variation to the archaeological hammerstones.

Overall, thirty-eight cobbles were provisioned to participants.

A large rectangular granite block (34cm x 15 cm x 13 cm; 16.7 kg) was provided to the participants. The granite block could be used by the participants as, for example, an anvil in bipolar technique or a passive hammer in the eponymous technique (*16, 26, 42-45*).

Safety Procedures

Safety procedures were designed and finalized based on the requirements of and consultation with authorities at the University of Tübingen.

During testing, all participants as well as the experimenter(s) were required to wear a pre-defined set of safety gear as follows (when applicable: gear type, brand, safety rating):

- Gloves (Galilee Royaltec CUT II), varying sizes, Category II
- Clear face shield (Clearways CV83P)
- Adjustable leather aprons (Babimax Retardant Welders Apron)
- Leather leg guards (AS Arbeitsschutz 272-302), welding standards
- Overshoe boots (Honeywell Over Shoes 971), varying sizes

Gloves and leather aprons protected individuals from cuts and percussive damage from shards of sharp materials. Category II gloves are a category above the minimal level for protection against

cuts and scrapes, like those involved in knapping. Leg and foot protection were also included to prevent damage (via cutting) to the legs and/or clothing of participants, and to prevent small flakes and debitage from falling into participants' trouser legs or shoes. The clear face mask prevented sharp flakes and debitage from injuring eyes, nostrils, or mouth.

For tests performed after the advent of the COVID-19 pandemic (i.e., starting with participant P4), participants were additionally required to wear disposable gloves under the cut-resistant gloves (available in three sizes) and face masks (disposable face masks were provided upon request if participants did not bring their own or wished to change masks mid-test). The clear face shield was disinfected with antimicrobial disinfectant spray before and after use at each session. The room was also ventilated for at least 30 minutes before and throughout testing. Participants were required to fill out a questionnaire regarding their symptomatic status, in compliance with university regulation and legal standards. Individuals who were symptomatic or recently exposed could not be tested. Pregnant women could also not be tested due to an inability to meet the official recommendations for maintenance of safe distance. It was not possible to carry out the originally intended longitudinal testing strategy (10 sessions totaling 22 hours), because of COVID-19-related restrictions and dropouts caused by the pandemic.

Although it was determined to be highly unlikely that injuries would occur given the safety gear set we used, further proactive measures were taken to ensure the safety of participants. The main experimenter (W.D.S.) completed first aid training prior to the beginning of testing. The course (DE. EHB Erste Hilfe Grundschulung) took place on December 8, 2018, at the Johanniter Unfall Hilfe e.V. in Tübingen, Germany. A first aid kit (DE. KFZ-Verbandkasten), purchased for the purposes of this study was present at all testing sessions in case of any injuries to the participants.

Due to health and safety concerns, in cases where participants spontaneously engaged in potentially dangerous behavior (e.g., attempting to lift heavy objects like the granite block, throwing objects), experimenters discouraged repetition of the behavior by informing the participants that any repetition would result in the premature cessation of the testing session for their dangerous conduct (with compensation only for the completed testing time). There were no cases in which a session was ended prematurely because of dangerous conduct, but there was one case (P18) of a session ending early since the safety equipment – specifically the face shield – could not be sufficiently fixed to the participant's head and therefore repeatedly fell to the ground, therefore preventing the safety protocol from being fulfilled (Table S2).

Under the above guidelines, throwing was considered potentially dangerous conduct in most cases. Though throwing was a coded behavior due to its relevance as a technique for creating usable flakes (e.g., as spontaneously developed by the enculturated bonobo Kanzi) (*38*), participants were discouraged from throwing after their first attempt, as it was deemed a health and safety concern. It was up to the experimenter's judgment whether a behavior qualified as "throwing" and therefore potentially dangerous, or "dropping" which was not considered as dangerous conduct. Generally, behaviors, even if then discouraged or disallowed, were still coded and recorded throughout the test.

Ethics and Data Protection

Approval from the Ethics Committee for Psychological Research, Faculty of Science, University of Tübingen for the proposal titled "Study on Human Stone Tool Making and Using" was received on July 15, 2019. The Committee reviewed the research methodology, including safety procedures, and data protection framework, as well as all relevant documents that were to be distributed to the participants in the study.

The data management and protection strategies and all participant documents were reviewed before the study by ZENDAS, the Data Protection Office for Universities in Baden-Württemberg. Full approval was received on June 12, 2019. The review process by ZENDAS had the following primary results as relates to the actual implementation of the study:

- The participant documents were edited to fit within the framework of the General Data Protection Regulation (GDPR) (EU) and the Landesdatenschutzgesetz Baden-Württemberg (LDSG) (EN. State Data Protection Law).
- Video recordings were required by ZENDAS to be pixilated to guarantee the anonymity of study participants. For this purpose, the video editing software Sensarea was applied, the usage of which was specifically approved by ZENDAS.
- ZENDAS determined that audio (i.e., voice recordings) can never be completely anonymized, even if with audio editing software. As a result, audio was not allowed to be recorded – ensured by us by physically blocking the microphone jack of the camcorders.

Study participants were given pseudonymized codenames, so that their real identities and personal information were never directly connected to study data.

<u>Participants</u>

Participants were recruited via online and local newspaper classified listings. Participants were requested to fall within the age range specific by the advertisements (18 to 55 years of age). Due to data protection concerns, we did not ask participants to directly report their age, instead only asking for the decade of birth in the pre-study demographic questionnaire. Participation in the study was contingent upon good eyesight or good corrected eyesight, right-handedness, unrestricted physical ability (i.e., the freedom to move arms/hands and strength to perform the task), no diagnoses for neurological or psychiatric illnesses, and no active psychoactive drug use;

participants were asked to self-filter according to these criteria prior to testing. Inclusion in the study was conditional upon the provision of informed consent, whereby participants were given sufficient time to read all information sheets before signing the relevant consent forms. We targeted the recruitment of an equal number of males and females.

Since we could not know *a priori* how many technique-naive (and totally naïve) participants we would encounter during data collection, we tested a relatively large sample of participants (thirty participants, before dropouts). Combined with the four-hour testing sessions, we hypothesized that this would prove sufficient to result in the logically necessary amount of data – i.e., that at least one participant would re-innovate at least one knapping technique during testing, while being naïve at time of innovation to this know-how (based on prior power analysis in (*40*) for a similar study on chimpanzees). Conducting these experiments is taxing and resource-intensive on several levels, meaning that testing further participants was considered logistically unsound (this would have been impossible anyway due to the repeated onset of further COVID-19-related restrictions).

Testing Apparatus and Procedure

Video recording of the testing sessions was performed using one Sony Handycam HDR-CX250 (Camera 2), one Sony Handycam HDR-CX330 (Camera 3), and one Sony Handycam HDR-CX450 (Camera 1), all mounted on tripods situated on spots pre-designated (with duct tape) in the testing space. Recording using the camcorders was done so at the widest possible angle setting for the lenses, and the centrally located camcorder (Camera 2) was enhanced with a wide-angle lens attachment to capture as much action as possible in the testing space. The whole experiment was recorded entirely without audio due to data protection constraints set by ZENDAS, the Central Data Protection Authority for Universities in Baden-Württemberg. The

microphone jacks for the camcorders were physically obstructed to ensure that no audio was recorded (this was once tested and verified to work before testing began).

The introduction to the apparatus included some clarification of the mechanics of the tendon box (i.e., that its rope prevents its door from being opened, but without using the words “rope” or “door”). The experimenter used gesturing and general language towards the glass blank, river cobble, and granite block – rather than specifically naming the objects. This means the participants were not verbally cued to the properties of the material or any specific potential uses for the objects. Experimenters therefore never provided any instruction about stone toolmaking or use, nor were participants allowed to elicit any help in this regard from the experimenters. Experimenters were required to follow a script to ensure the same vague terminology and phrasing was used for all participant sessions.

Upon each successful solving of the problem, the participant gained access inside the puzzle box to a reward-token - a paper slip representing the monetary reward (printed on one side). New reward tokens were placed inside the box after each success, and the box then re-set. The first reward value was always 10 €, but values varied thereafter. Collected reward values across all successes were added together at the end of the session and the sum then paid out to the participant in legal tender.

The determination of the reward value in each session and with each attempt followed a formula of diminishing returns, since the initial motivation was assumed to be high and, in an ecological setting, the first “portion(s)” of a raw material or food source would likely be the most valuable and additional retrievals (and the effort involved) would be decreasingly profitable over time. The curve to the diminishing returns formula that was used has a long tail (with low value rewards) as the overall success rate of participants could not be predicted and the total money

available for each session (per the research grant ERC Starting Grant STONECULT) was limited. Participants were guaranteed to earn 10 € for their first success. Afterwards, the new reward would be selected from the deck of tokens and then placed face-down in the rope apparatus (only revealed to the participant after they succeed). Each time a token was drawn from the deck of notes, a note with 0,01€ was added into the deck and the full deck was shuffled (thus reducing the expected reward value with each iteration; participants were not made aware this was occurring). The reward "deck" contained the followed distribution of rewards:

- 10 € x 1
- 2 € x 10
- 1 € x 20
- 0,50 € x 40
- 0,10 € x 50
- 0,01 € x ∞ (theoretically only)

Exhaustion of cores applied in cases where participants stopped engaging with the test material (e.g., actively percussing or pounding the glass or stone, pursuing repeated or new solutions for the tendon box apparatus) for more than five minutes at a time. If and when a participant exhausted a core, they were provided a new one by the experimenters. Participants were allowed a maximum of one new blank for each hour of the experiment (e.g., if a participant received their second core at one hour and twenty minutes into the session, they were allowed to receive a new core - at the earliest - two hours and twenty minutes into the study).

If participants deemed their final core exhausted before the end of the last hour of the study, they had the option of waiting idly (receiving full reimbursement for the hour) or of leaving (receiving only money for the time they waited and forgoing any additional reward, even if their core was not truly exhausted). This particular situation did not come to fruition in the study.

This study involved three participant questionnaires that were given out at the end of testing.

All participants – regardless of testing outcome – received the questionnaire aimed at determining their past experiences, if any, with stone tools and toolmaking (a "naivety check") (DE. *Fragebogen zu den Vorerfahrungen der Studienteilnehmer*, EN. *Questionnaire on the Past Experiences of Study Participants*). We used this (unique) approach (i.e., providing *post*-test questionnaires), in order to prevent conveying any know-how information about stone tools and/or knapping techniques before testing the participants for their spontaneous tool making skills (therefore preserving their know-how naivety, i.e., in cases where they were indeed naïve). See section 9 for original German text and English translation.

Participants who, during their entire test, did not make their own cutting tool at all or who made cutting tool(s) but did not use them were given a questionnaire aiming to determine the cause of their lack of success (DE. *Post-Studie Fragebogen (von Teilnehmern, die nicht erfolgreich waren),* EN. *Post-Study Questionnaire (for participant who were not successful)*). This questionnaire was in two parts: an initial section and a follow-up immediately after the participants were provided information on the goal of the task and stone tools. See below for original German text and English translation.

A third questionnaire was given specifically to participants who, during their entire test, created at least one cutting edge but failed to utilize these to open the tendon box (DE. *Weitere Fragen im Falle von einer Teillösung der Aufgabe*, EN. *Additional questions in case of a partial solution*). See below for original German text and English translation.

<u>Data</u>

The behaviors were separated into bouts, which were either single actions or sequences of actions with a recorded start and end point. The start of a bout was defined by the engagement of the participant with the raw materials and puzzle box in any way that might lead to toolmaking

or opening of the box. The end of a bout was defined when the character of behavior changed (thus starting a separate, new bout, e.g., the participant fluidly switched from passive hammer technique to bipolar technique) or the participant ceased engaging in the particular behavior for thirty seconds or longer.

Coding of toolmaking techniques was determined through an “elements-based” approach (adapted from previous approaches) (*68*), which was determined as a reliable way to define the behaviors actually performed by the study participants (since many of these behaviors did not readily fit in pre-existing classificatory models of EST production techniques) while also being agnostic to both the mechanic and outcome of any fracture events. This approach was also conceived as applicable to further scenarios involving humans and non-human animals, making cross-context and cross-species comparisons more viable. In this outcome-neutral approach, there are four classes of elements that can be present in the constellation of objects and actions that take place in toolmaking: active, passive, auxiliary, and target elements. Active elements are objects in motion (i.e., imbued with/possessing kinetic energy), and passive elements are the primary recipient of force from an active element. Auxiliary elements are primarily used for stabilization or support to a passive element but can also be transformed by the toolmaking process or even be mechanically active in a secondary sense to the active element. Target elements are, in essence, the target of a tool use behavior (e.g., the rope of the tendon box or in ecological scenarios of extractive foraging: nuts or animal carcasses).

The identification of these elements was used to categorize early knapping techniques (*16, 26, 42-45*) as: freehand knapping, passive hammer technique, bipolar technique, and projectile technique (with additional coding for simultaneous toolmaking and use).

In *freehand knapping*, the agent (here, study participant) uses an object held in one hand (an active element) to induce a possible transformation in another object that is held in the other hand and/or supported against the body of the agent (a passive element or a second active element).

Passive hammer technique involves one object held by the agent (an active element) being moved (mainly) to be transformed by another (passive element) that is either stationary on the floor or even the floor itself (note that passive elements can also be transformed in this scenario).

Bipolar technique involves one object (active element) held and moved by the agent to induce a potential transformation in another (passive element), which is stationary and resting on/supported by another object (auxiliary element).

Projectile technique involves one object (active element) being dropped onto (i.e., leaving body contact) or thrown toward another object or the floor (passive element).

Simultaneous toolmaking and tool use can involve a similar constellation of elements to the previous categories (in this study, only passive hammer technique was not a technique for TMU), but also the target element, meaning it is the simultaneous production of a tool or cutting edge and use of a tool.

Toolmaking mechanics were coded as being percussion, friction, or pressure, while outcomes were coded in terms of whether there was fracture of an object and which object was fractured in the bout.

A hypothesis-unaware person (J.K.) was hired as a student research assistant with the assignment of performing secondary coding of a subset of the data (all behavioral clips from 7 participants,

25% of participant sample). The reliability coder used the same coding protocol as the experimenter W.D.S. Furthermore, this individual was untrained in the methods and theory of lithic technology and thus considered as unbiased towards traditional methods of understanding toolmaking and the techniques thereof.

Participants were ranked for their naivety on the basis of their responses to the *Questionnaire on the Prior Experience of Study Participants*. We used the following ranks (categories) to characterize the amount and nature of experience individuals had with stone tools:

0. Participant is totally naive
1. Participant has conceptual knowledge of stone tools (i.e., conscious of their existence)
2. Participant has seen a stone tool or a depiction of a stone tool
3. Participant has seen the production of a stone tool [*here*, technique know-how could have been imparted on participants]
4. Participant has direct hands-on experience with knapping and, with it, at least one knapping technique

Labels contained the code name of the participant, the number of the blank (labelled sequentially from 1 to *n*), and whether it was successful or unsuccessful to access a reward.

Detached pieces were identified as either flakes or angular fragments. We followed the same definition used by (*13*), whereby flakes show all the hallmarks of conchoidal fracture (i.e., platform and bulb of percussion) while angular fragments were those artefacts that lacked hallmarks of conchoidal fracture.

The validity of our claims regarding knapping depends upon the accurate identification of the artefacts as flakes (i.e., produced by conchoidal fracture in the same manner as the prehistoric equivalents). Typing of detached pieces was performed by W.D.S. and then repeated by a hypothesis-unaware second observer (A.F.). A.F. is an experienced lithic analyst, but – as alluded to – was not informed of the purpose of the typing and the theoretical and methodological framework of the experiments. The second observer was tasked with identifying a randomly selected subsample of 155 detached pieces (approximately 10% of the 1553 detached pieces from all participants) as either flakes or angular fragments.

For enhanced clarity about the categories, illustrations of toolmaking behaviors were created in Adobe Illustrator by tracing over screen captures of video recordings.

Lithic illustrations are a standard practice in archaeological research and analysis (*79*). Cores were covered in non-reflective spray (due to transparency and reflectiveness of glass) and scanned using an ARTEC Spider while flakes were photographed with a digital camera, as attempts at 3D scanning produced an unclosed mesh with noise along the fine, cutting edge of the glass flakes. 3D scans of cores were imported into Blender, converted into orthographic projection (this mapped the technological information of the 3D cores onto 2D space without distortion) and then screen-capped (using an internally developed protocol serving as a "digital photography studio"). Attributes of the artefacts were traced in Adobe Illustrator to generate lithic illustrations according to the STIVA method (*79*).

Supplementary results: behaviors and toolmaking techniques

Three out of the 28 participants did not produce and/or use cutting tools within the first 2 hours of the testing session. Therefore, according to the predesigned protocol, at this point these participants received the "Impossible Flake". All three participants used the impossible flake as a tool to then access the reward. Afterwards, one of these three participants (P6) produced and used glass cutting edge, while the two remaining participants of these three (P21 and P23) produced glass cutting edge but then failed to use it as a tool to open the tendon box. Of the latter two, P21 reported in the PSQ that he recognized that a cutting edge was made but felt there were other solutions worth attempting, while P23 reported in the PSQ that she failed to recognize that she had created any useable glass cutting edge.

Another participant (P2) produced an angular fragment from the granite brick and used it to sever the rope but was not able to repeat this or other techniques and instead opted to pursue other means of opening the tendon box.

Confirmed toolmaking events were more stereotyped and less variable in terms of their technique categorization, with five individuals using one technique exclusively (Tables S6 and S9). 12 individuals used two of the different technique categories to successfully fracture objects, while 9 individuals used three of the different technique categories, and one individual implemented all four technique categories for toolmaking. However, the difference in distributions of technique preferences between potential and confirmed toolmaking was not statistically significant (Pearson's chi-squared test, X^2=6.3808, p=0.1725).

The first potential toolmaking event was most often categorized as freehand technique (n=10, 35.7%) followed by both passive hammer and bipolar (for each: n=7, 25.0%) and then projectile (n=4, 14.3%) (Table S5). The distribution shifted when considering only events with successful

fractures, with bipolar technique as the most common (n=10, 37.0%), followed by passive hammer and freehand (for each, n=8, 29.6%) and projectile again the least common (n=1, 3.7%). The difference in distributions between potential and confirmed toolmaking, however, was not statistically significant (Pearson's chi-squared test, X^2=2.601, p=0.4573).

Interrater reliability using Cohen's kappa tests determined that video coding of toolmaking and techniques (Table S8) in the selected subsample of trials (n=228) was reliable between the two observers using both counts of relevant bouts in each trial ($\kappa > 0.6$) and presence/absence of relevant bouts in each trial ($\kappa > 0.9$).

Supplementary results: material outcomes

Only 2 individuals did not produce any objects that could be classified as flakes (P2 and P23), though in both these cases, the glass hemispheres showed signs of removals by conchoidal fracture. Most individuals produced assemblages that could be characterized as flake-dominated (i.e., >50% of artefacts were conchoidal flakes) (Table S10). There were a total of 1172 flakes (73.3%), 361 angular fragments (22.6%), 33 glass cores (2.1%), and 33 river cobbles (as cores, percussors, or hammer-cores; 2.1%).

More explicit details on the material outcomes of toolmaking and more exhaustive technological analysis will be reported in W.D.S., J.S.R., & C.T. *in prep*.

The hypothesis-unaware second observer (A.F.) typed 122 of 155 (78.71%) of the detached pieces as flakes, compared with 126 flakes out of 155 artefacts (81.29%) for the original observer, W.D.S. The second observer identified 7 objects as flakes that the original observer did not, two of which were non-glass objects. The original observer identified 11 objects as flakes (all made of glass) that the second observer identified as angular fragments. The Cohen's kappa test determined that the flake identification protocol was reliable in the selected subsample (κ = 0.637).

There were no statistical differences in terms of EPA between any naivety groups and between all naivety groups and the Oldowan reference material (Table S11).

There were no statistical differences in terms of PD between the naivety groups from the experiment. However, each pairwise comparison for PD between experimental naivety groups and the Oldowan reference material was significant (Table 12).

All pairwise comparisons used a Wilcoxon rank sum test, and no *p*-value adjustment was applied.

Questionnaire on the Prior Experiences of Study Participants

All participants were given the Questionnaire on the Prior Experience of Study Participants. This questionnaire was given to the participants after their testing was complete in order to not compromise the integrity of the study and the naivety of the individuals (*pre*-test questionnaires would have compromised these). Responses to this questionnaire were used to determine the naivety level of each participant.

Fragebogen zu den Vorerfahrungen der Studienteilnehmer<br>Datum: _________<br>Teilnehmer ID: __________<br>Hinweis: Ihre Antworten, egal wie diese ausfallen, werden keine negativen Folgen für Sie haben. Sie können sämtliche monetären Belohnungen („Preise“) sowie sämtliche Aufwandsentschädigungen, die Sie bisher bekommen haben, behalten.<br>Bitte beantworten Sie folgende Fragen möglichst knapp, oder kreisen (falls diese Optionen vorhanden ist) die passende Antwort ein.<br>1. Sind Sie derzeit an einer Universität eingeschrieben? Falls ja, für welchen Studiengang?<br><br>2. In welchem Semester befinden Sie sich?<br><br>3. Falls Sie kein Studierender sind, was ist Ihr höchster Schulabschluss? Wenn Sie zu einem früheren Zeitpunkt an einer Universität eingeschrieben waren, bis zu welchem Semester hatten Sie studiert?<br><br>4. Haben Sie jemals an Kursen teilgenommen , in denen Steinwerkzeuge zumindest *erwähnt* wurden?<br>O JA O NEIN<br>a. Falls ja, beschreiben Sie bitte entsprechende Kurse (und den Veranstaltungsort). Gehen Sie bitte insbesondere die Art und Tiefe der Informationen, die dort über Steinwerkzeuge vermittelt wurden ein, v.a. bezüglich der Art und Weise der Herstellung von Steinwerkzeugen.<br><br>b. Falls ja, wann haben Sie an diesem Kurs/diesen Kursen teilgenommen (z.B. „vor dieser Studie, im Jahre XYZ“)? [Mehrfachnennung möglich]?<br><br>5. Haben Sie bereits an *Studien* zu Steinwerkzeugen (z.B. zu deren Beschreibung oder Herstellung) teilgenommen?<br>O JA O NEIN<br>a. Falls ja, bitte die Studie(n) im Folgenden kurz beschreiben (und Studien-Ort bitte angeben). [Mehrfachnennung möglich]	*Questionnaire on the Prior Experience of Study Participants*<br>Date: _________<br>Participant ID: __________<br>Note: Your answers, regardless of how they turn out, will lead to no negative consequences for you. You can keep all monetary rewards (prizes), along with all the hourly compensation, that you have thus far received.<br>Please answer the following questions as succinctly as possible, or cross out the suitable answer (if this option is provided).<br>1. Are you currently enrolled at a university? If yes, in which degree of study? If no, please continue with question 3.<br>2. In what semester are you currently?<br>3. If you are not a student, what is your highest attained degree? If you were enrolled at a university at an earlier point in time, up to what semester had you studied? What did you study?<br>4. Did you take part in a course, in which stone tools were at minimum mentioned?<br>O YES O NO<br>a. If yes, please describe the corresponding course(s) (and the course setting). Please provide especially the type and depth of information about stone tools that was conveyed there, above all as to the ways of stone tool production.<br>b. If yes, when do you take part in this course (e.g., “Before this study, in the year XYZ”)? [Multiple answers possible]?<br>5. Have you already participated in studies on stone tools (e.g., on their description or their manufacture)?<br>O YES O NO<br>a. If yes, please briefly describe them (and provide the setting/study site). [Multple answers possible]<br>b. If yes, when did you take part in this study/these studies (e.g., “Before this study, in the year XYZ”)? [Multiple answers possible]?

b. Falls ja, wann haben Sie an dieser Studie/diesen Studien teilgenommen (z.B. „vor dieser Studie, im Jahre XYZ“)? [Mehrfachnennung möglich]?<br><br>6. Haben Sie schon einmal an einem/mehreren praktischen Kursen teilgenommen, deren Ziel die Herstellung von Steinwerkzeugen war?<br>O JA O NEIN<br>a. Falls ja, beschreiben Sie bitte kurz entsprechende Kurse (und den Veranstaltungsort).<br><br>b. Falls ja, wann haben Sie an diesem Kurs/diesen Kursen teilgenommen (z.B. „vor dieser Studie, im Jahre XYZ“)? [Mehrfachnennung möglich]<br><br>7. Haben sie sich jemals aktiv über Steinwerkzeuge informiert oder passiv etwas über Steinwerkzeuge gelernt (z.B. durch Bücher, Radio, Fernsehen, Internet, Museumsbesuch etc.)?<br>O JA O NEIN<br>a. Falls ja, bitte geben Sie an, welche Art des Medium (z.B. Radio). [Mehrfachnennung möglich]<br><br>b. Falls ja, wie detailliert waren diese Informationen (z.B. Zeitleiste der Steinwerkzeuge; Art und Weise der Herstellung etc.)? [Mehrfachnennung möglich]<br><br>c. Falls ja, wann haben Sie diese Information passiv oder aktiv erhalten? (z.B. „vor dieser Studie, im Jahre XYZ“)? [Mehrfachnennung möglich]<br><br>8. Haben Sie schon einmal Abbildungen (Illustrationen, Fotografien, Videos, Diagramme, Museumsaustellungen, etc.) die frühe Steinwerkzeuge zeigen, gesehen?<br>O JA O NEIN<br>a. Falls ja, bitte beschreiben Sie kurz (und, wenn Sie wollen, zeichnen) Sie, was Sie gesehen haben. [Mehrfachnennung möglich]<br><br>b. Falls ja, wann haben Sie diese zum ersten Mal gesehen (z.B. „vor dieser Studie, im Jahre XYZ“)? [Mehrfachnennung möglich]<br><br>9. Haben Sie sich jemals den *Prozess* der Steinwerkzeugherstellung angesehen, also die Art und Weise der Herstellung (einschließlich live, YouTube –Videos, Dokumentarfilmen etc. – oder auch nur als Illustrationen (Bilder oder Video))?<br>O JA O NEIN<br>a. Falls ja, bitte beschreiben Sie, was Sie gesehen haben (v.a. Detailgrad der Werkzeugherstellung) und wo. [Mehrfachnennung möglich]	6. Have you already taken part in one or more practical courses, in which the goal was the production/manufacture of stone tools?<br>O YES O NO<br>a. If yes, please briefly describe them (and the course setting) [Multiple answers possible]<br>b. If yes, when did you take part in this course/these courses (e.g., “before this study, in the year XYZ”)? [Multiple answers possible]<br>7. Have you ever actively informed yourself about stone tools or passively learned about stone tools (e.g., via books, radio, television, internet, museum visits, etc.)?<br>O YES O NO<br>a. If yes, please indicate the kind of medium (e.g., radio). [Multiple answers possible]<br>b. If yes, how detailed was this information (e.g., a timeline of stone tool types; ways of stone tool production, etc.)? [Multiple answers possible]<br>c. If yes, when did you receive this information? (e.g., „Before this study, in the year XYZ“)? [Multiple answers possible]<br>8. Have you already seen depictions of early stone tools (illustrations, photographs, videos, diagrams, museum exhibitions, etc.)?<br>O YES O NO<br>a. If yes, describe briefly describe (and, if you want, draw) what you have seen. [Multiple answers possible]<br>b. If yes, when did you see this/these for the first time (e.g., “Before this study, in the year XYZ”)? [Multiple answers possible]<br>9. Have you ever viewed the process of stone tool manufacture, like the manner of production (including live, YouTube videos, documentaries, etc. – also as just illustrations (pictures or videos))?<br>O YES O NO<br><br>a. If yes, please describe what you have seen (above all, the level of detail of the stone tool production) and where. [Multiple answers possible]<br>b. If yes, when do you see this for the first time (e.g., “Before this study, in the year XYZ”)? [Multiple answers possible]<br><br>10. Have you ever received instructions of stone tool making?<br>O YES O NO<br>a. If yes, please describe what kind of instruction this was (above all, the level of detail of the stone tool production) and where you received it. [Multiple answers possible]<br>b. If yes, when did you receive this instruction (e.g., “Before this study, in the year XYZ”)? [Multiple answers possible]

b. Falls ja, wann haben Sie dies zum ersten Mal gesehen (z.B. „vor dieser Studie, im Jahre XYZ“)? [Mehrfachnennung möglich]<br>10. Haben Sie jemals Anleitungen zur Steinwerkzeugherstellung bekommen?<br>O JA O NEIN<br>a. Falls ja, bitte beschreiben Sie, welche Art von Anleitung dies war (v.a. Detailgrad der Werkzeugherstellung) und wo Sie diese erhalten haben. [Mehrfachnennung möglich]<br><br>b. Falls ja, wann haben Sie zum ersten Mal diese Anleitungen erhalten (z.B. „vor dieser Studie, im Jahre XYZ“)? [Mehrfachnennung möglich]<br><br>11. Würden Sie von sich sagen, dass Sie in generellem Bezug auf Steinwerkzeuge generell gute Kenntnisse haben – in dem Sinne, dass Sie diese Kenntnisse außerhalb unserer Studie gewonnen haben?<br>O JA O NEIN<br>12. Würden Sie von sich sagen, dass Sie in Bezug auf die *Herstellung* von Steinwerkzeuge gute Kenntnisse haben – in dem Sinne, dass Sie diese Kenntnisse außerhalb unserer Studie gewonnen haben?<br>O JA O NEIN<br>13. Haben Sie das Gefühl, das Sie vor der Studie relevante Informationen zu Steinwerkzeugen und/oder zur Steinwerkzeugenherstellung besaßen, die durch die obigen Fragen nicht abgedeckt sind?<br>O JA O NEIN<br>a. Falls ja, bitte beschreiben Sie, welche Art von Informationen (v.a. Detailgrad der Werkzeugherstellung) und wo Sie diese erhalten haben. [Mehrfachnennung möglich]<br><br>b. Falls ja, wann haben Sie diese Informationen erhalten (z.B. „vor dieser Studie, im Jahre XYZ“)? [Mehrfachnennung möglich]<br><br>14. Haben Ihnen irgendwelche Information, die Sie vor der Studie erhalten haben, in dieser Studie geholfen Steinwerkzeuge herzustellen?<br>O JA O NEIN<br>a. Falls ja, bitte beschreiben Sie, welche Art von Informationen (v.a. wie viel Detail zur Werkzeugherstellung) und wo Sie diese erhalten haben. [Mehrfachnennung möglich]<br><br>b. Falls ja, wann haben Sie diese Informationen erhalten (z.B. „vor dieser Studie, im Jahre XYZ“)? [Mehrfachnennung möglich]<br>15. Gibt es weitere Informationen zu Ihrer Person , die Sie eventuell für relevant für uns/Steinwerkzeuge betrachten?<br>O JA O NEIN<br>a. Falls ja, bitte unten beschreiben:	11. Would you yourself say, that you generally have good knowledge in relation to stone tools – in the sense that you earned this knowledge outside of our study?<br>O YES O NO<br>12. Would you yourself say, that you have good knowledge in relation tot he specific means of production of stone tools – in the sense that you earned this knowledge outside of our study?<br>O YES O NO<br>13. Do you have the feeling that you possessed or received relevant information to stone tools or stone tool production before or during the study that was not covered by the questions above?<br>O YES O NO<br>a. If yes, please describe what kind of information (above all, the level of detail of stone tool production) and where you received this. [Multiple answers possible]<br>b. If yes, when did you receive this information (e.g., “Before this study, in the year XYZ)? [Multiple answers possible]<br><br>14. Did you receive any information before or during this study, which helped you produce stone tools in this study?<br>O YES O NO<br>a. If yes, please describe what kind of information (above all, how much detail of stone tool production) and where you received this information. [Multiple answers possible]<br><br>b. If yes, when did you receive this information (e.g., “Before this study, in the year XYZ”)? [Multiple answers possible]<br><br>15. If there any more information about you that you potentially consider relevant for us/stone tools?<br>O YES O NO<br>a. If yes, please describe below:

Post-study Questionnaire (for participants who were not successful)

This questionnaire was given only to participants who, during the entire test, did not succeed at the task in the sense of creating and using cutting tools.

Post-Studie Fragebogen (von Teilnehmern, die nicht erfolgreich waren)<br>*Teil 1*<br>Datum: _________<br>Teilnehmer ID: __________<br>Bitte beantworten Sie die folgenden Fragen.<br>Hinweis: Ihre Antworten, egal wie diese ausfallen, werden keine negativen Folgen für Sie haben.<br><br>1. Was war Ihrer Meinung nach das Ziel unseres Experiments – was, glauben Sie, haben wir versucht zu untersuchen?<br><br>2. Was glauben Sie, hat Sie vom erfolgreichen Lösen der Aufgabe abgehalten? Beschreiben Sie bitte kurz die Faktoren, die Sie dabei womöglich gehindert haben.	*Post-Study Questionnaire (for Participants who were not successful)*<br>*Part 1*<br>Date: ________<br>Participant ID: _________<br>Please answer the following questions.<br>Notice: Your answers, regardless of how they turn out, will not result in negative consequences for you.<br><br>1. What, in your opinion, was the purpose of our experiment – what, in your belief, did we attempt to research?<br><br>2. What do you think prevented you from a successful solution of the task? Please briefly describe the factors that have possibly hindered you from doing so.
Post-Studie Fragebogen (von Teilnehmern, die nicht erfolgreich waren)<br>*Teil 2*<br>*(Die Teilnehmer bekommen zuvor eine Kopie von der „Nachbesprechung der Problemlösestudie".)*<br><br>Datum: _________<br>Teilnehmer ID: __________<br><br>Beantworten Sie bitte kurz die folgenden Fragen und denken Sie an die Information, die Sie gerade erhalten haben („Nachbesprechung der Problemlösestudie").<br><br>1. Denken Sie, dass Sie das Ziel des Experiments vollständig, oder nahezu vollständig, verstehen?<br>O JA O NEIN<br>2. Glauben Sie, im Hinblick auf die eben erhaltenen Informationen, dass noch andere Faktoren Sie vom erfolgreichen Lösen der Aufgabe abgehalten haben? Beschreiben Sie bitte kurz die Faktoren, die Sie dabei vermutlich besonders gehindert haben.	*Post-Study Questionnaire (for Participants who were not successful)*<br>*Part 2*<br>*(The participant receives beforehand a copy of the "Problem-solving Study Debriefing")*<br>Date: ________<br>Participant ID: _________<br><br>Please briefly answer the following questions and think about the information that you have just received ("Problem-solving Study Debriefing").<br><br>1. Do you think that you understand the purpose of the experiment completely or nearly completely?<br>O YES O NO<br>2. Do you think, thinking back on the information you just received, that yet more factors prevented you from successful solution of the task? Please briefly describe the factors that have probably hindered you from doing so.

Additional questions in case of a partial solution

This questionnaire was given only to participants who, during the entire test, did not fully succeed at the task in the sense that, while they created cutting edge on glass and/or stone, they never *used* the created cutting edge to overcome the rope of the tendon box.

Weitere Fragen im Falle von einer Teillösung der Aufgabe<br>Datum: _________<br>Teilnehmer ID: __________	*Additional questions in case of a partial solution*<br>Date: _________<br>Participant ID: __________
Bitte beantworten Sie die folgenden Fragen.<br>Hinweis: Ihre Antworten, egal wie diese ausfallen, werden keine negativen Folgen für Sie haben.	Please answer the following questions.<br>Note: Your questions, regardless of how they turn out, will lead to no negative consequences for you.
Das Ziel dieses Experiments, also die vollständige Lösung der Aufgabe, beinhaltete a) die Erschaffung von Objekten mit scharfen Kanten, alleinig durch entsprechende Verwendung der bereitgestellten Materialien, und b) das Nutzen dieser scharfen Kanten, um Zugang zu den Belohnungen zu erhalten (also durch Durchschneiden von einem Seil oder einer Membran).	The goal of this experiment was twofold a) the creation of sharp edges via the usage of the provided materials, and b) the cutting of either the rope or the membrane of the apparatuses, in order to receive the reward(s).
Sie bekommen diesen Fragebogen, weil Sie zwar ein Objekt (oder mehrere Objekte) mit solchen scharfen Kanten erschaffen haben, aber diese Kanten dann nicht genutzt haben, um an die Belohnungen zu gelangen.	You have created one or more objects with sharp edges, but you did not see the task all the way through to the finish.
1. Haben Sie während des Tests erkannt, dass Sie scharfe Kanten erschaffen haben?<br>O JA O NEIN	1. Did you recognize that you had created such an objects or objects with sharp edges?<br>O YES O NO
a. Falls ja, warum haben Sie (Ihrer Meinung nach) diese scharfe Kanten nicht verwendet, um mindestens einen von den zwei Apparaten zu öffnen und die enthaltene Belohnung zu bekommen?	a. If yes, why did you not use such sharp edges, in order to open one of the apparatuses and receive the reward?
2. Was glauben Sie, hat Sie vom erfolgreichen Lösen der Aufgabe abgehalten? Beschreiben Sie bitte kurz die Faktoren, die Sie dabei womöglich gehindert haben.	2. What do you believe prevented you from a successful solution of the task? Please briefly describe the factors that possible would have stopped you.
Vielen Dank für das Ausfüllen dieses Fragebogens. Bitte denken Sie daran, alle Informationen zur Studie vertraulich zu behandeln und diese nicht anderen Personen weiterzugeben.	Thank you for filling out this questionnaire. Please bear in mind to handle all information about this study in confidentiality and to not pass on this information to any other person.

Falls Sie weitere Fragen in Bezug auf das Experiment stellen möchten, kann der Versuchsleiter diese nun gerne beantworten.<br>Falls Sie später weitere Fragen haben, können Sie diese an William Snyder schicken.<br>Vielen Dank für Ihre Teilnahme!	If you have further questions in relation to the experiment that you wish to pose, the lead experimenter can gladly answer those now.<br>If you have more questions later, you can send these to William Snyder.<br>Thank you for your participation!

Experimenter script

In order to maintain the same method for each participant session, the experimenter followed a standardized script for each test, to prevent experimenter error and to prevent variation in information given across participants. Icons were included to make for quick and easy accessibility of the relevant responses to specific situations. Thus, the script is presented here in the same exact form as the printout used by the experimenter.

Key phrases that need to be standardized to ensure that all participants get the same info:

Pre-test preparations:

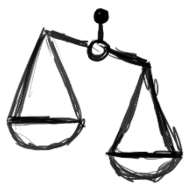

Understanding of pre-study documents, incl. participant information and consent

- Haben Sie alle Dokumente, die Sie von mir per Email bekommen haben, komplett gelesen und verstanden? Sie können mir jetzt Fragen stellen, die Sie noch über die Dokumente haben.
    - EN: Have you completely read and understood all documents, that you received before by Email? Please pose any questions you have about the documents now.

If *no* to previous question:

- Bevor Sie teilnehmen dürfen, müssen Sie alle Dokumente durchlesen, und Ihnen durch Unterschrift zustimmen.
    - EN: Before you may participate, you must now read through all documents, in order to know and clearly understand your rights and the requirements of this study.

Security measures

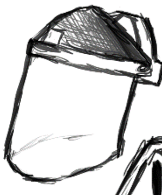

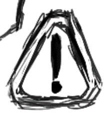

- Bevor Sie in den Testraum hineinkommen dürfen, müssen Sie sich zuerst (jedes Mal!) diese Schutzausrüstung anziehen. Sie müssen alle Teile anziehen. Wenn diese Ausrüstung während des Tests unangenehm wird oder irgendwas wehtut, informieren Sie mich bitte. Bevor Sie die Schutzausrüstung ausziehen dürfen, müssen Sie erst den Testraum verlassen. Durch die Schutzausrüstung ist es zwar sehr unwharscheinlich, dass Sie sich verletzen, aber falls Sie sich in irgendeiner Weise trotzdem verletzen sollten, informieren Sie mich bitte sofort.
    - EN: Before you may enter the testing space, you must first (and every time) put on the safety gear. You must put on every piece of this gear. If you are uncomfortable during the experiment or something hurts, please inform me immediately. Before you can remove the safety gear, you must first leave the testing space. It is also important that you inform me of injuries of any kind (within the session).
- Falls möglich, sollten Sie Schmuckstücke von Ihrer Händen abziehen. Ich bitte Sie auch, mir ihr Handy zu geben. Aber keine Angst: Ihr Handy und ihr Schmuck werden auf dieses Bord in eine Box gelegt und bleiben dort – für Sie sichtbar - bis zum Ende der Sitzung. Sie dürfen Ihr Handy während der Testsitzung nicht nutzen (ich empfehle, es nun lautlos zu schalten). Dies ist nötig, weil wir Sie auf Ihre eigene Lösungsansätze testen, und deswegen müssen wir verhindern, dass Sie nach Lösungsansätzen im Internet suchen.
    - EN: If possible, you should remove any jewelry from your hands. I also ask that you give me your phone. But no worries: Your phone and jewelry will be placed on this shelf in a box – visible to you - and will stay there until the end of the session. You may not use your phone during the session (I recommend to set it to

silent now); we are testing for your own individual solving approaches, and therefore, we want to prevent that you search for outside (=Internet) solutions.

Beginning:
[Before continuing, press record on all cameras and clearly show the coding sheet to each].

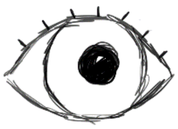

- Sobald ich “Start” sage, dürfen Sie anfangen. Wenn ich „Stopp“ sage, halten Sie bitte sofort inne (was auch immer Sie in dem Moment tun, müssen Sie dann stoppen).
    - EN: When I say Start, you may begin. When I say Stop, you must immediately stop (whatever you are doing, you must absolutely stop)
- Sehen Sie hier diese Aufgabe [point to the apparatus]? Bei *dieser* Aufgabe [tendon box] hindert Sie *dieser* Teil [point to rope] daran *diese* Tür zu öffnen um an die Belohnung zu gelangen. **Beachten Sie bitte die *anderen* Teile der Aufgabe *gar nicht*. Mit anderen Worten: konzentrieren Sie sich ausschließlich auf diesen [point] Teil der Aufgabe.** Zu Ihrer Information: Es *ist* möglich, diese Aufgabe mit Hilfe der Ihnen zur Verfügung gestellten Objekte zu lösen.
    - EN: Do you see this task here? With this task, this part [the rope] prevents you from opening this door and accessing the reward. Do not pay any attention to the parts of the apparatus. In other words: concentrate exclusively on this part of the task. For your information: it is possible to solve this task with the help of the objects that are placed for your availability.
    - Possible replies:
        - Meinen Sie diese Schnur/diese Seile/usw.?
            - EN: Do you mean this string/rope/ etc.?
            - Confirm, but do not repeat the term they use (refer to them always as “Teil”)
- Sie können sich jetzt gerne die Aufgabe anschauen. Wollen sie? [if yes, allow. If no, continue]
    - EN: You can now look at the apparatus. Would you to do so?
- Sie können alle diese Objekte [point to everything, except the apparatus] anheben und aufnehmen. Die Aufgabe selbst müssen Sie allerdings stehenlassen.
    - EN: You can lift and pick up all of these objects. You must leave the apparatus itself as it is.
- Sie können *alle* diese Objekte [point slowly at granite, hammerstones and glass blank] benutzen um die Aufgabe zu lösen, in jeglicher Weise - und ganz wie Sie es für richtig halten.
    - EN: You are allowed to lift all objects in the testing space except for the apparatus. You may use all of these objects in any fashion and as you see fit in order to solve the task.
- Es gibt *mehrere* Methoden, mit denen man die Aufgabe lösen kann. Einige Methoden dürfen wiederholt angewendet werden; andere dürfen nicht wiederholt werden. Ich werde Sie jeweils informieren, falls eine Methode *nicht* wiederholbar ist.

    - EN: There are several methods, with which one can solve the task. Some methods may be repeatedly used; others cannot be repeated. I will inform you each time, if a method is not repeatable.
- Sehen Sie die Belohnungen / das Monopoly-Geld? (point to apparatus and wait for an affirmative)
    - EN: Do you see the rewards? (point to apparatus and wait for an affirmative)
- Versuchen Sie, die Aufgabe zu öffnen, um die jeweils enthaltene Belohnung zu bekommen – diese Belohnung wird Ihnen am Ende der Sitzung in Echtgeld ausbezahlt (das heißt, *zusätzlich* zu ihrem generellen Aufwandsentschädigung pro Stunde)
    - EN: Attempt to open this apparatus, in order to be paid for the reward inside – this will be paid out to you at the end of the session in real money (in addition to the base hourly rate).
- Um die Videoaufnahmen zu synchronisieren werde ich nun kurz einen einzelnen Lichtblitz auslösen. Ich empfehle Ihnen kurz die Augen zu schliessen oder wegzuschauen. Auf drei löse ich den Blitz aus. 1, 2 , 3.
    - EN: In order to synchronize the video recordings, I will trigger shortly a single photo flash. I recommend that you briefly close your eyes or look away. On three I will trigger the flash. 1, 2, 3.
- Start, oder: Sie dürfen gerne anfangen. [Experimenter starts stopwatch and timers]
    - EN: Start, or: You may begin.

During Testing:

If they ask at any point what they can and cannot do:

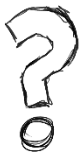

- Sie können mich zwar generelle Fragen stellen aber ich empfehle Ihnen das nicht zu tun. Ich darf Ihnen sowieso keine Lösungen nennen und die Antworten könnten Sie zudem verwirren. Deshalb: Probieren Sie am Besten einfach aus, was Ihnen einfällt. Ich würde Sie dann schon stoppen.
    - EN: You can ask me general questions but I recommend that you don’t. I am not allowed to name any solutions, and the answers could confuse you. As such: simply try out, whatever crosses your mind. I would stop you [if it is not appropriate].
- Kann ich X, Y, Z tun?
    - EN: Can I do X, Y, Z?
    - Sie können alles versuchen, mit Ausnahme von allen jenen Methoden bei denen ich dazusagte dass Sie sie nicht mehr machen dürfen (also: XXX [list the ones that apply at that moment, starting with „Ihnen sind bisher noch keine Methoden verboten worden“]).
        - EN: You can use any method, with the exception of all methods that I said to you that they are no longer allowed to be done. (also: XXX [list the ones that apply at that moment, starting with “For you, there are so far no methods that have been forbidden”
- Kann ich/darf ich X [the stones, the core, anything other than the boxes of the apparatus] zerbrechen?
    - EN: Can I/am I allowed to break X?

- Probieren Sie einfach das aus, was Ihnen einfällt. Ich würde Sie dann schon stoppen.
  - EN: Simply try out, whatever comes to mind. I would stop you [if it is not appropriate].

If they insist:

- Alles was ich Ihnen sagen kann ist: Sie können alle Objekte benutzen die vor Ihnen liegen, um die Aufgabe zu lösen.
  - EN: You can use all objects that are lying in front of you to solve the apparatus.

If they are vocally angry, frustrated, confused, etc.:

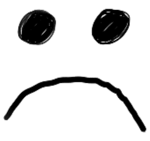

- Der Versuchsaufbau erlaubt mir nicht Ihnen Tips oder Anleitungen zu geben.
  - The experimental setup does not allow me to give you tips or instructions.
- If they are frustrated about changes: Bitte erinnern Sie sich daran, dass die Teilnehmerinformation folgenden Satz enthielt: „Der Versuchsleiter behält sich vor, die Regeln während des Versuchs zu vervollständigen oder abzuändern, um einen ordnungsgemäßen Versuchsablauf zu sichern."
  - EN: Please also recall that the Participant Information sheet contains the following phrase: „The investigator reserves the right to amend or modify the rules during the trial in order to ensure proper experimental procedure and protocol."

Additionally, offer general encouragement when the participant is struggling to dissuade loss of motivation.

- [zum Beispiel]: Sie können gerne weitermachen. Sie haben noch Zeit. Machen Sie daher ruhig weiter, und probieren Sie einfach.
  - EN: You can gladly continue. There is still time. You still have time. Steadily continue and simply try things out.

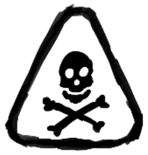

If they engage in dangerous behaviour or attempt to lift the anvil above the ground:

- Stopp. Machen Sie bitte XXX nicht [whatever halts their dangerous actions]- Eine Wiederholung davon wird zu der Beendung dieser Sitzung und keiner weiteren Teilnahme von Ihnen führen.
  - Stop. Do not do XXX. Repetition thereof will lead to the ending of this session and no further involvement of you in the study.
- Stop. Bitte dieses Objekt nicht anheben.
  - Stop. Please do not lift up this object [granite anvil].

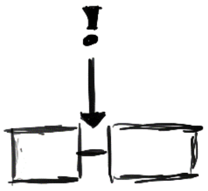

If they continue [more than 5 minutes in total] to explore the outside of the apparatus with their hands or objects (including but not limited to: touching and trying to turns screws, touching and hitting the non-solution-related parts of the apparatuses, trying to pull on the door of the tendon box, etc.):

- Stop. Bitte halten Sie kurz inne. Ich möchte Sie noch einmal erinnern [point to the apparatus and repeat the description].
  - EN: Stop. Please cease briefly. I would like to remind you once more the description of the apparatuses.

Repeat as necessary:

- Bei dieser Aufgabe [tendon box] hindert Sie dieser Teil [point to rope] daran diese Tür zu öffnen um an die Belohnung zu gelangen. Beachten Sie nicht den Rest der Aufgabe gar nicht. Zu Ihrer Information: Es *ist* möglich, diese Aufgabe mit den Ihnen zur Verfügung gestellten Objekten zu lösen.
    - EN: With this task, this part prevents you from opening the door and accessing the reward. Do not pay any attention to the rest [of the apparatus]. For your information: it is possible to solve these tasks with the objected that are placed for your availability.

If they use a method besides cutting (after they complete the method once):
If they rub on the rope:

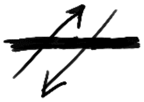

- Ab jetzt dürfen Sie nicht mehr die Aufgabe durch Reiben lösen. Reiben ist nun keine erlaubte Lösung mehr, denn das nicht der Lösungsansatz an dem wir interessiert sind. Versuchen Sie also weiterhin eine Lösung zu finden.
    - EN: Henceforth, you may no longer solve the task by rubbing. Rubbing is no longer an allowed solution, as it is not the solution that we are interested in. Please continue to attempt to find another solution. [Note: Here and for other 'unwanted' solutions, we include this specific phrasing due to Pilot Participant 2 mistakenly interpreting the goal of the experiment to be diversity of solutions and therefore potentially self-directing their behavior to accomplish this.]

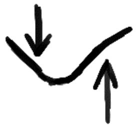

If they push down or up with a blunt surface or edge:

- Ab jetzt dürfen Sie nicht mehr die Aufgabe durch Pressen oder Ziehen lösen. Pressen oder Ziehen sind nun beides keine erlaubte Lösung mehr, denn das nicht der Lösungsansatz an dem wir interessiert sind. Versuchen Sie also weiterhin eine Lösung zu finden.
    - EN: Henceforth, you may no longer solve the task by pressing down or pulling up. Pressing is no longer an allowed solution, as it is not the solution that we are interested in. Please continue to attempt to find another solution.

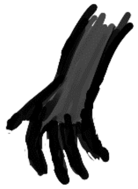

If they use their hands to pull or push open an apparatus:

- Ab jetzt dürfen Sie die Aufgabe nicht mehr ohne Zurhilfenahme von Objekten lösen. Die Lösung ohne Objekte ist nun keine erlaubte Lösung mehr, denn das nicht der Lösungsansatz an dem wir interessiert sind. Versuchen Sie also weiterhin eine Lösung zu finden.
    - EN: Henceforth, you may no longer solve the task without the help of the objects. The solution without objects is no longer an allowed solution, as it is not the solution that we are interested in. Please continue to attempt to find another solution.

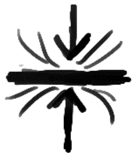

If they open the apparatus by knocking together two objects:

- Ab jetzt dürfen Sie die Aufgabe nicht mehr durch Aufklopfen lösen. Aufklopfen ist nun keine erlaubte Lösung mehr, denn das nicht der Lösungsansatz an dem wir interessiert sind. Versuchen Sie also weiterhin eine Lösung zu finden.
    - EN: Henceforth, you may no longer solve the task by knocking together two objects. This knocking is no longer an allowed solution, as it is not the solution that we are interested in. Please continue to attempt to find another solution.

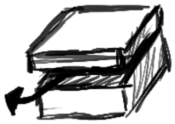

If after two hours they are not successful and are to receive the Impossible Flake:

- Bitte nehmen Sie dieses Objekt [the impossible flake]. Sie dürfen es einmalig benutzen, um die Aufgabe zu lösen. Danach wird es Ihnen wieder weggenommen.
  - EN: Take this thing/object. You may use it once to solve the task. Afterward, it will be taken away.

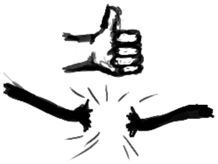

When they succeed at cutting:

- Stopp! Halten Sie bitte inne. Danke. Bitte legen Sie nun das Ding in Ihrer Hand auf den Boden. Ich benötige nun ein wenig Zeit, um Daten aufzunehmen. Setzen Sie sich bitte derweil auf den Stuhl dort in der Ecke, bis ich Ihnen sage das sie zurückkehren können.
  - EN: Stop! Please cease [what you are doing]. Thank you. Lay the thing in your hand [=flake or core-tool] on the ground. I require a little time to record data. Sit down please meanwhile on the chair in the corner, until I say that you can return.

First time: explain the rule that successful tools are to be transferred to the experimenter including reference to statement of new rules in consent form

- DE: Bitte erinnern Sie sich, dass die Teilnehmerinformation folgenden Satz enthielt: „Der Versuchsleiter behält sich vor, die Regeln während des Versuchs zu vervollständigen oder abzuändern, um einen ordnungsgemäßen Versuchsablauf zu sichern.“ Ab jetzt gilt nun die folgende Regel: Jedes Mal, wenn Sie die Testaufgabe lösen, müssen Sie das Ding, mit dem Sie die Aufgabe gelöst haben, mir übergeben.
  - EN: Please recall that the Participant Information sheet contains the following phrase: „The investigator reserves the right to amend or modify the rules during the trial in order to ensure proper experimental procedure and protocol.” From now on, the following rule applies: every time that you solve the task, you must hand over to me the thing with which you have solved it.

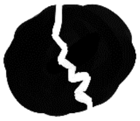

If they shatter the core producing multiple pieces:

- Stopp! Halten Sie bitte inne. Bevor Sie weitermachen dürfen, müssen Sie nun ein Teilstück wählen, mit dem Sie weitermachen wollen. Alle anderen Teile werden von mir eingezogen.
  - EN: Stop! Please pause. Before you are allowed to continue, you must now select one piece with which you want to continue working. All other pieces will be confiscated by me.

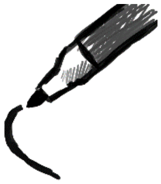

Upon creation of a core-tool and use of edge on core-tool to cut apparatus:

- Stopp! Halten Sie bitte inne. Bitte bleiben Sie genau so wie Sie gerade sind. Ich werde nun mit einem Stift den Bereich markieren, den Sie gerade genutzt haben, um die Aufgabe zu öffnen. Bitte beachten Sie die neue Regel: Markierte Bereiche dürfen Sie nicht mehr nutzen um den Aufgabe zu öffnen.
  - EN: Stop. Please cease what you are doing. Please stay exactly as you are now. I will mark the edge that you just used to solve the apparatus with a pen. Please be aware of the new rule: marked edges may no longer be used to open the apparatus.

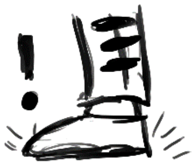

After the first success, extra safety instruction due to sharp materials on ground:

- Für Ihre eigene Sicherheit knien Sie bitte von nun an nicht mehr auf dem Boden. Auf dem Boden können sich Splitter befinden.
    - EN: For your own safety, please do not kneel on the ground from now on. Sharp chips can be found on the ground.

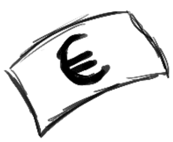

After the first success:

- Dieser Ausdruck repräsentiert das von Ihnen gerade zusätzlich gewonnene Geld, also ihre Belohnung. Jedes Mal, wenn Sie die Aufgabe lösen, wird die jeweilig enthaltene Belohnung dort [in einer Box] gesammelt. Am Ende der Sitzung wird der Wert von allen Belohnungen zusammengerechnet. Sie werden dann die Gesamtsumme in Echtgeld erhalten (das heißt, sie bekommen sowohl die stündlichen Aufwandsentschädigungen als auch die gesammelten Belohnungen).
    - EN: This paper printout represents the money you have additionally won, otherwise your reward. Every time that you solve the task, the reward received thus far will be collected here. At the end of the session, the value of the rewards all together will be calculated. You will be paid the real money in one lump sum (that means, you receive both the hourly compensation and the rewards for successes).
- Bitte beachten Sie, dass ab jetzt die Ausdrücke nicht mehr lesbar ohne die Öffnung der Aufgabe werden (die Schriftseite wird nach unten gelegt). Die genaue Höhe der Belohnung entdecken Sie erst, wenn Sie die eder erfolgreich gelöst haben. Beachten Sie bitte: Die Höhe der Belohnungen wird ab jetzt durch eine Tombola [if they don‘t understand: „durch gemischte Karten“ or per „Lotterieverfahren“ or „zufällig“] festgelegt. Die Zettel wurden vor der Sitzung von mir gemischt (d.H., randomisiert). Der Höchstwert der zukünftigen Belohnungen liegt bei 10 €.
    - EN: Be aware that from now on the paper printouts will no longer be legible without the opening of the apparatus (they will be placed face down). You will discover the value of the reward first when you successfully solve the task again. Please be aware: the values of the rewards will now be determined by a raffle/draw [if they don't understand: "by mixing of cards" or per lottery or "chance"]. The slips of paper were already shuffled by me before the session (i.e., randomized). The maximum value of future rewards is 10€.

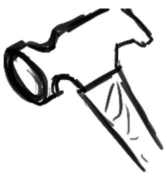

If the participant breaks the hammerstone, including gradually or in one event, there is a protocol to be followed. 1/3 volume lost (as visually judged by experimenter) elicits a question to the participant. ½ volume lost means automatic replacement of hammerstone.

- Stopp. Ich kann Ihnen anbieten, dieses Objekt [point to hammer] gegen dieses [point to potential replacement] auszutauschen.. Möchten Sie es austauschen?
    - EN: Stop. I can offer you this: you can trade this object for this. Would you like to trade?

- Stopp. Dieses Objekt [point to current hammer] muss nun gegen dieses [point to new hammer] ausgetauscht werden.
  - EN: Stop. This object must now be traded out for this.

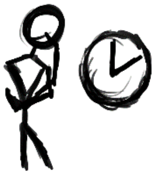

If they stop attempting to make flakes are or are unsuccessful at making a flake for an extended period of time (=min. of 5 minutes), they have already made flakes with this core, **and** more than an hour of testing has passed by. They cannot request a new core before they have intentionally made flakes on the core. Researcher's judgment of intentional flaking and core depletion is applied here.

- Hindert Sie etwas daran weiterzumachen?
  - EN: Is something preventing you from continuing?
- If they say yes [in the sense towards the problem of not being able to work the core further] and have made flakes: Sie können ein neues Objekt bekommen [hold up new core]. Sie können ein solches, neues Objekt alle 60 Minuten erhalten, falls Sie dann ein neues Objekt wünschen. Beachten Sie, falls Sie ein neues Object bekommen wollen, wird Ihnen jeweils das alte Objekt von mir abgenommen. Falls 60 Minuten seit der letzten Objektannahme noch nicht vergangen sind, müssen Sie warten bis diese 60 Minuten rum sind.
  - EN: You can receive a new object. You can receive such a new object every 60 minutes, in case you wish for one. Be aware, in case you do want to receive a new object, the old object will, in each case, be taken away by me. If 60 minutes have not yet passed since the last handout, you must wait until this 60 minutes is finished.
- [At the end of the study if they exhausted the core and want to end:] Sie befinden sich in der letzten Stunde der Sitzung. Falls sie nicht weitermachen können, und trotzdem ihre volle zeitliche Aufwansentschaedigung erhalten wollen, müssen Sie horbleiben und warten bis zum Ende der Testsitzung. Sie dürfen naturlich auch jederzeit gehen, aber Sie bekommen dann kein Geld für die restliche Zeit der Sitzung. Sie haben dann natürlich auch keine Möglichkeit mehr, weitere Belohnungen aus der Aufgabe zu erhalten.
  - EN: You are now in the last hour of the session. In case you cannot continue, and yet still want to receive your full compensation, you must stay and wait until the end of the session. You may of course also leave at any time, but you will receive no money for the remaining time of the session. You then have also no more opportunity to obtain further rewards from the apparatus.

<u>Artefact label</u>

The following label was printed and filled out for each tool, core, core fragment and debitage collection.

Participant: ______________________

Session #: _______________________

Date: ___________________________

Core #: __________________________

Not from core? (i.e., HS or AN)

____

Artefact #: ________________________

Success # (if relevant): _________________

Type (circle): Flake-tool *S* *U*

Core-tool

Core fragment

Core

Debitage collection

Other: __________________

Live coding sheet

The following represents the live coding sheet that was filled out as a paper copy by the experimenter during the sessions and later used as a reference for formulating the video coding of behaviors.

Coding Sheet # ____/____

HIDE FROM PARTICIPANTS AT ALL TIMES

Date	ID	Gender	Session	Core #	Start Time	End Time
__ __ 20__		M / F				

	Passive Hammer	Bipolar	Free-handed	Assisted Free-handed	Intent. throw/drop
Attempt=X					
Time					

	Friction/rubbing	Pushing with blunt surface	Pushing with sharp edge	Cutting
Attempt=X				
Time				

Time	Impossible Flake	Technique						Outcome		Use				Core	Follow-up needed
		1		2	3	4	5	6	7	8		9		10	
		Passive hammer		Bipolar	Free-handed	Assisted free-handed	Intent. throw/drop	Flake	Edge on core-tool	Cutting of rope		Other method		Core exhaustion	
		C	E							S	U	S	U		

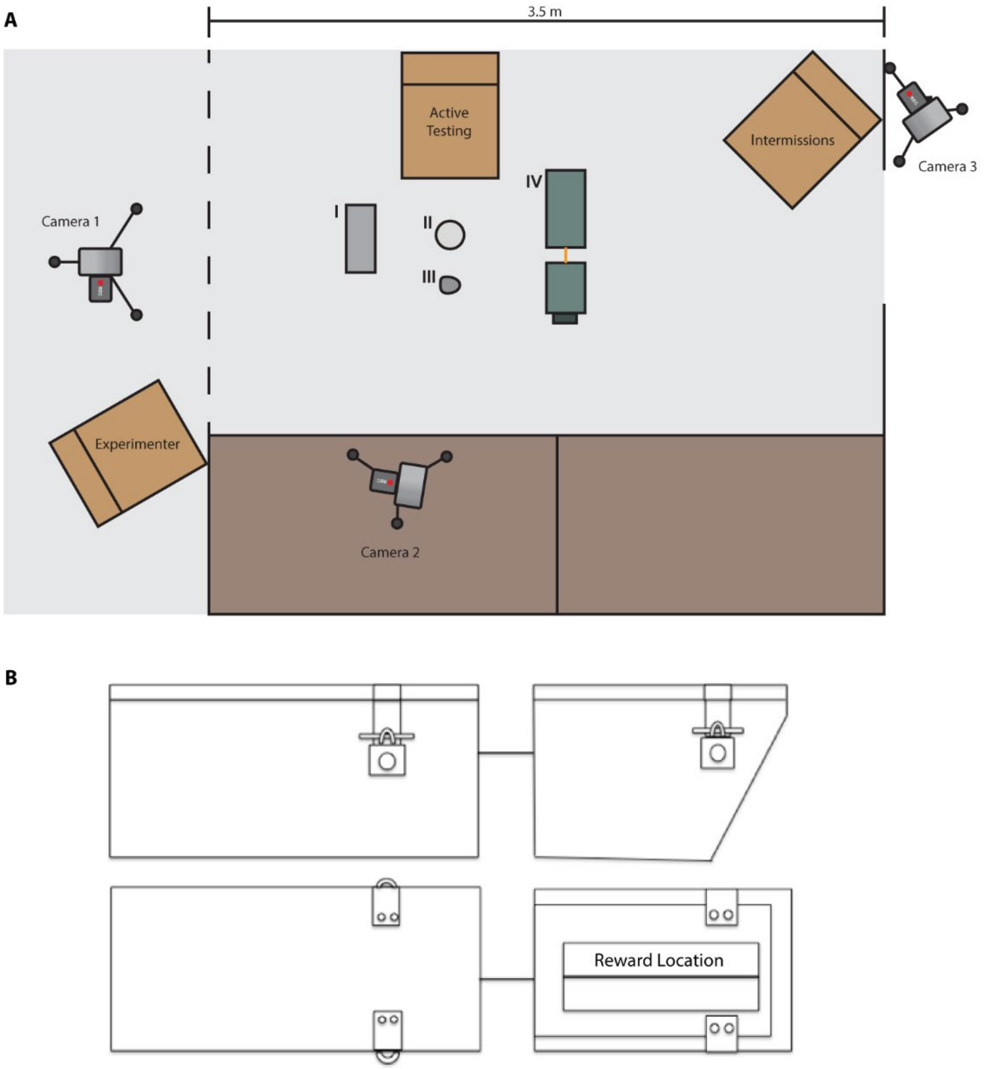

Fig. S1. The experimental apparatus. (**A**) Basic layout of the experiment, including the main testing materials like the (**I**) granite block, (**II**) glass hemisphere, (**III**) river cobble, and (**IV**) puzzle box. (**B**) Schematic of the puzzle box with side (top) and top (bottom) views.

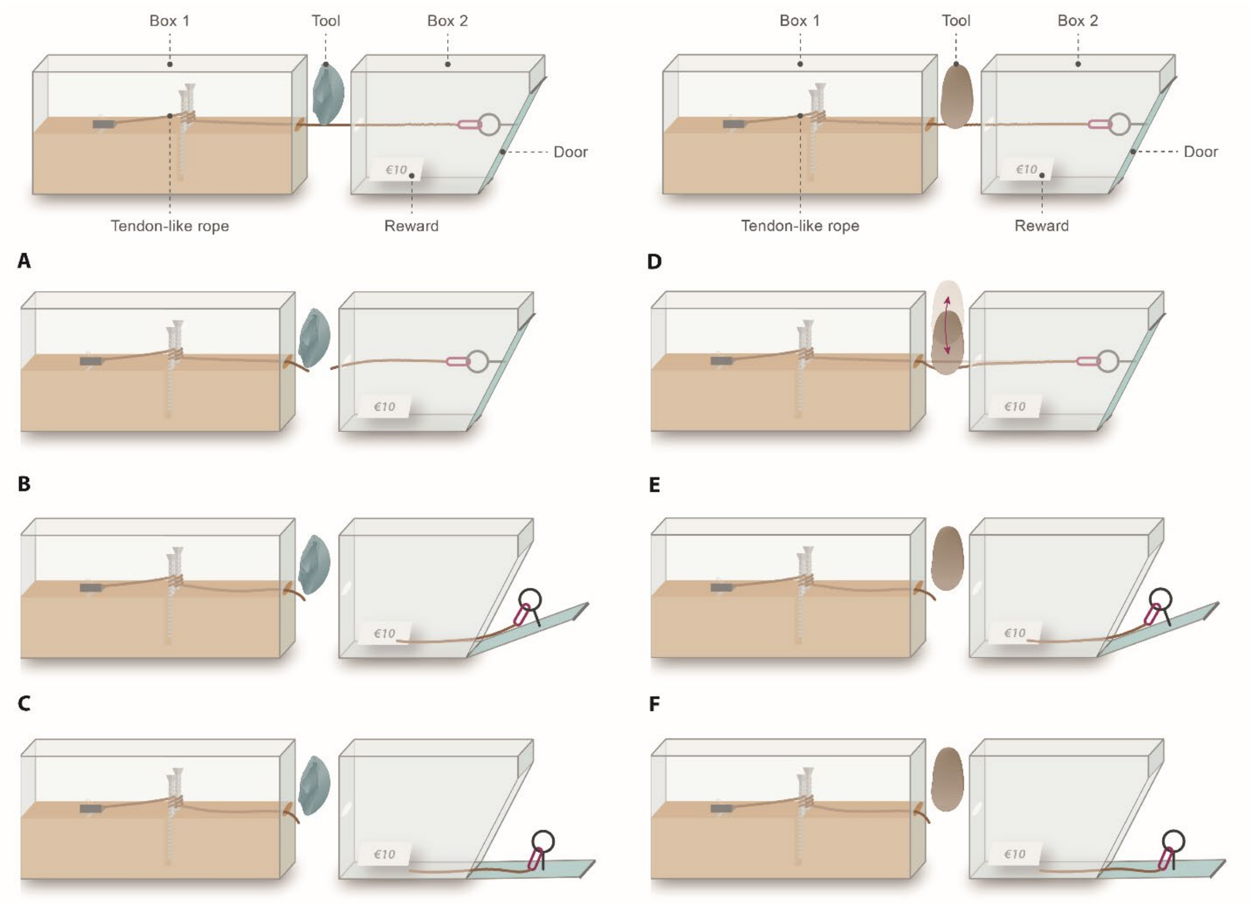

Fig. S2. Alternative pathways to solving the puzzle box. Cutting with a flake-tool (Fig.3) is not the only potential solution for the puzzle box. Here are two examples of said alternative solutions. In the first example (**A** to **C**), cutting edge created on the core (i.e., a core-tool) is used to cut the rope and therewith make the reward accessible by opening the puzzle box. In the second example (**D** to **F**), a non-cutting solution is applied. In this case, the river cobble is used to hammer on the rope with a percussive action until the rope breaks, thus opening the door and making the reward accessible to the participant. Some other examples of non-cutting solutions that participants applied included rubbing with a blunt edge (e.g., the river cobble or the round face of the glass hemisphere), picking apart the rope threads using their fingers, and stepping on top of the rope.

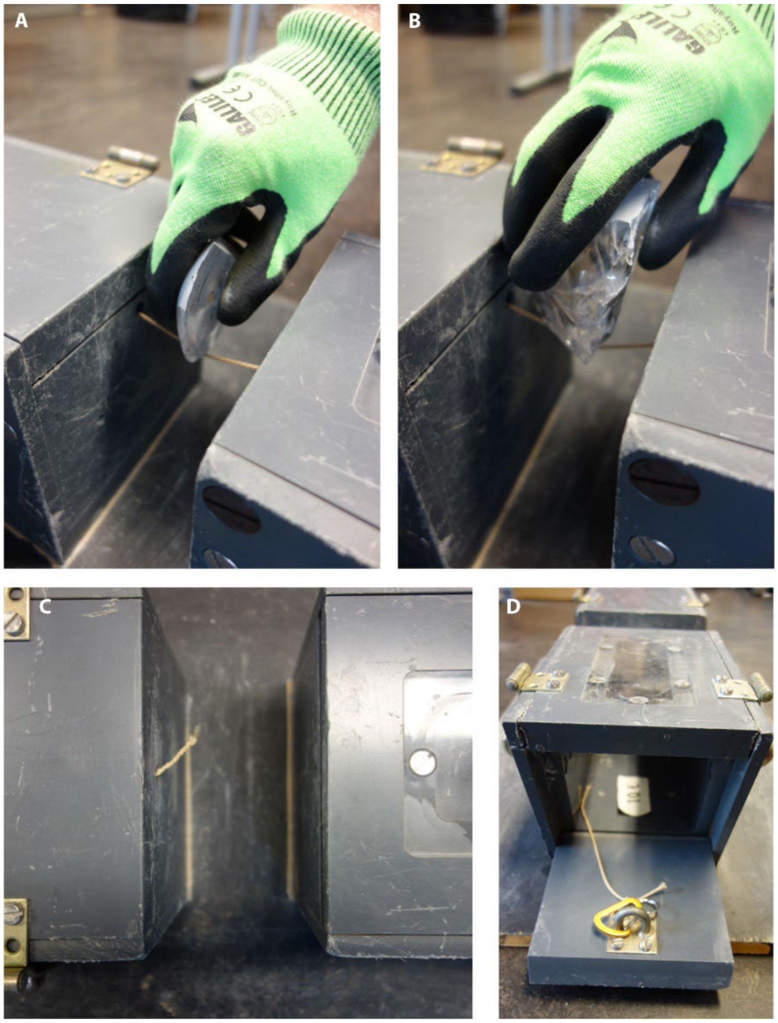

Fig. S3. Cutting tool use. (**A**) The use of a glass cutting tool (flake) to sever the rope and thereby open the puzzle box. (**B**) The use of a glass cutting tool (here, a core-tool) to sever the rope and open the puzzle box. (**C** to **D**) Opening of the puzzle box as a consequence of the tool use action. (**C**) Breakage of the puzzle box rope. (**D**) Opening of the freed door of the front compartment, allowing access to the reward token. pictures are derived from re-enacting by the experimenter, in order to demonstrate the use of a tool and the mechanism of the puzzle box with clarity that could not be achieved from the captured video stills of the actual tests. Photo Credits: Claudio Tennie and William D. Snyder, University of Tübingen.

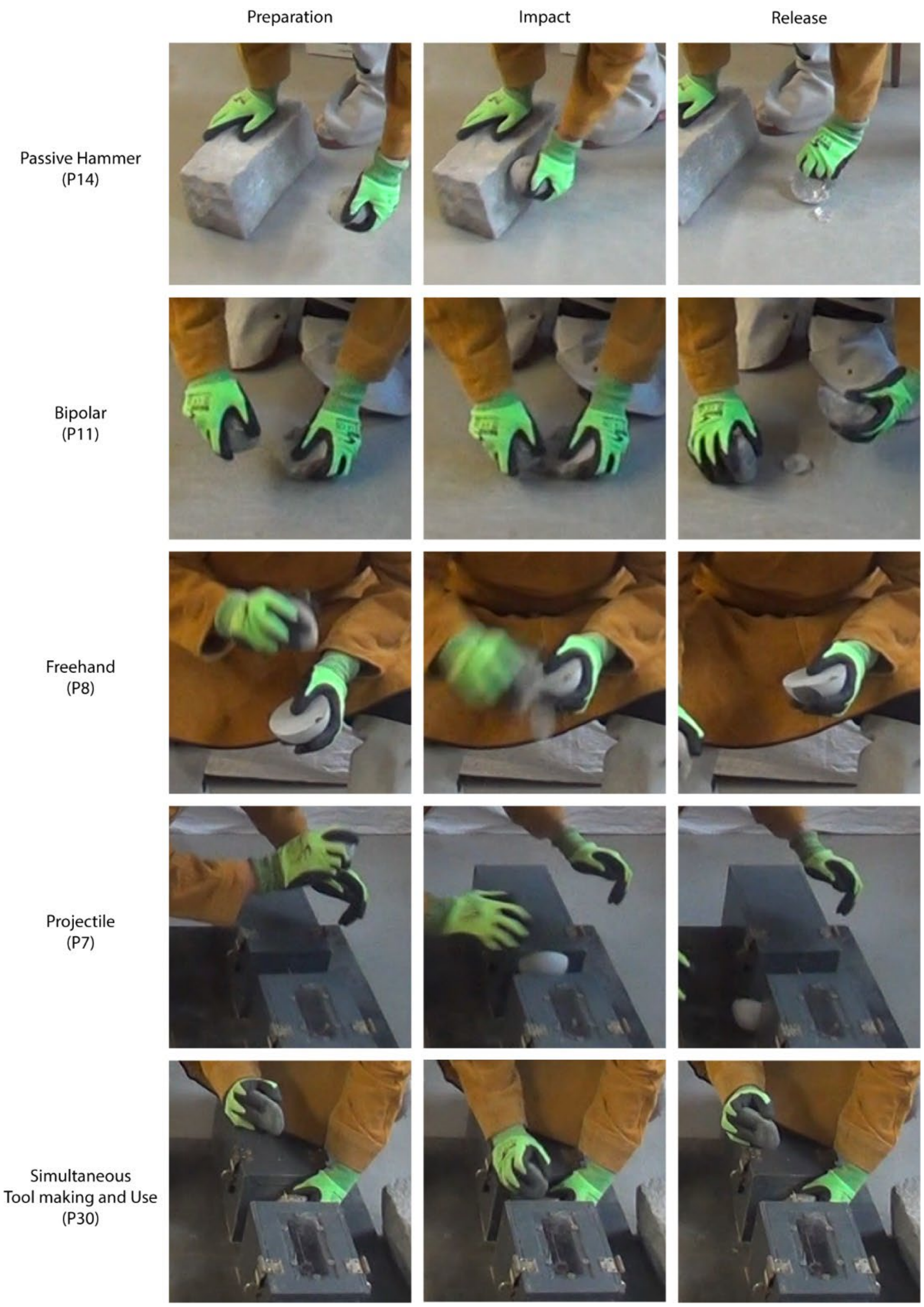

Fig. S4. Still frames of tool making technique action sequences. Top row, passive hammer technique (performed by P14, Trial 33). Second row, bipolar technique (performed by P11, Trial 41). Third row, freehand technique (performed by P8, Trial 2). Fourth row, projectile technique (performed by P7, Trial 2; no fracture occurred). Bottom row, simultaneous toolmaking and use, freehand technique (performed by P30, Trial 68). Video Still Credits: William D. Snyder, University of Tübingen.

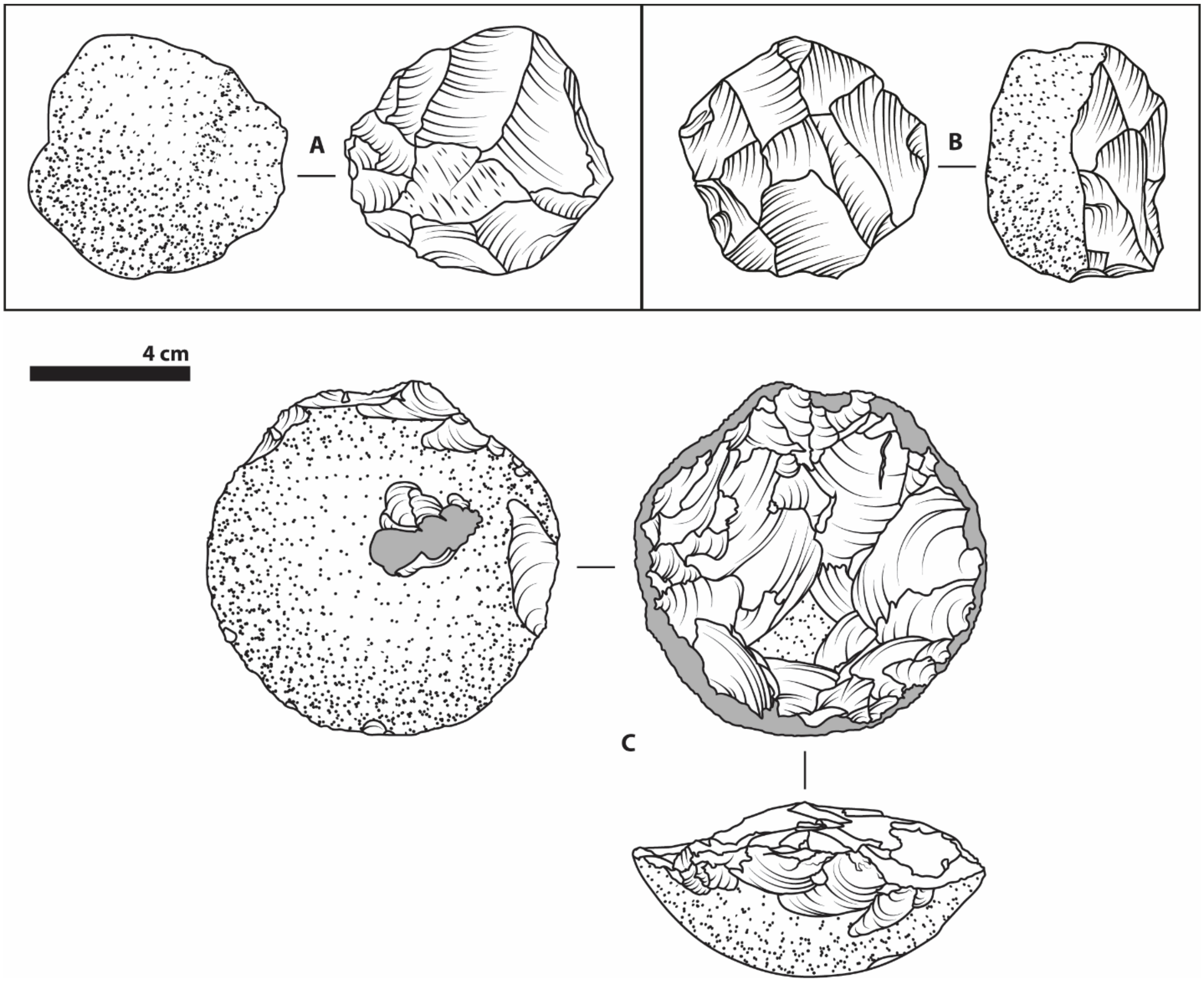

Fig. S5. Radial, or centripetal, reduction. The second most frequent core morphology from this study, after multifacial cores, were radial, or centripetal, cores. Here are two archaeological examples with radial knapping sequences: a "partial discoid" from Gona, Ethiopia dated to approximately 2.6-2.5 Ma (*73*) and "unifacial bipolar flaking" on obsidian (volcanic glass) from Melka Kunture, Ethiopia, where exploitation of obsidian during the Oldowan started around 1.7 Ma (*55*). These archaeological examples are shown in comparison with a selected similar core, which was produced by a totally naïve participant in this study (P14).

Table S1. Demographic and experiential information.

Individual	Sex	Decade of Birth	Naivety Level
P1	F	1990s	3
P2	F	1990s	2
P3	M	1990s	2
P4	F	1990s	2
P5	M	2000s	2
P6	F	1990s	2
P7	M	2000s	1
P8	M	1990s	2
P9	M	1960s	2
P10	F	1970s	2
P11	F	1990s	0
P12	F	2000s	2
P13	M	1970s	2
P14	M	1980s	0
P15	M	1990s	2
P16	M	1970s	2
P17	F	1960s	2
P19	F	2000s	4
P21	M	2000s	2
P22	M	1990s	2
P23	F	2000s	2
P24	M	1980s	2
P25	F	1980s	2
P26	F	1990s	2
P27	M	1990s	2
P28	F	1960s	3
P29	F	1960s	2
P30	M	2000s	2

Table S2. Unsuccessful and excluded participants. *QUP* is the Questionnaire for Unsuccessful Participants, which was given in two parts to those participants that failed to make *and* use cutting tools. *PSQ* is the questionnaire titled “Additional questions in case of a partial solution”, which was given to participants that created cutting tools but failed to use them to open the puzzle box. Here, we report the full answers, translated from German to English. Participants highlighted in grey were those whose data were excluded from the analyzed datasets.

Individual	Performance	Result	QUP Part 1	QUP Part 2	PSQ
P2	Produced an angular fragment from granite and used it for cutting the rope, but subsequently resorted to other apparatus solution techniques	Failure to reproduce the initial toolmaking success, participant given QUP	1: “If humans in our century are capable of using stone tools, or rather can come to the same approaches as in the time that these objects were the only tools”<br><br>2: “Too many own thoughts, if a new idea is again rubbing (or instead maybe cutting). Not attempted, if I myself thought that it is really nothing new”	1: “Yes”<br><br>2: “Yes, the thought that I should find a new approach. Rather thought about physics than on the use of objects. The thought that everything I used would be taken away, I thought ultimately that I must also come to a solution without the objects”	n.a.
P6	Provided impossible flake, subsequently produced and used glass cutting tools	Treated as success, so not given QUP or PSQ	n.a.	n.a.	n.a.
P21	Provided impossible flake, produced viable cutting tools but did not use them to open tendon box	Failure to use participant-created cutting tools, participant given QUP and PSQ	1: “The behavior of humans by the solving of difficult tasks.”<br><br>2: “Creativity, i.e. to get the door open with many different options. Possibly, that there are still other solutions, e.g. to not sever the rope”	1: “Yes”<br>2: “Infrequent use of stones, thus it was difficult to spontaneously make a "tool"”	Realized that sharp-edged objects (i.e. glass flakes) had been produced but did not used them because “[they] already had used many options to cut apart the rope”
P23	Provided impossible flake, produced viable cutting tools but did not use them to open tendon box	Failure to use participant-created cutting tools, participant given QUP and PSQ	1: “How people deal with unfamiliar situations, for which they may have a hard time finding solutions"<br><br>2: “Inexperience with practical work and insecurity due to perplexity”	1: “Yes”<br><br>2: “In the course of schooling learned things were often repeated or rather applied, yet the problem solving competence was more rarely expected”	Did not realize that sharp-edged objects (i.e. glass flakes) had been produced and felt as if “[their] ideas were exhausted and also through further reflection, no new ideas arose”
P18	Safety concern	Session ended, data excluded	n.a.	n.a	n.a
P20	Experimenter error	Data excluded	n.a.	n.a	n.a

Table S3. Hammerstone weights from Olduvai Gorge Oldowan sites (*68*) and this study.

Site Name	Mean mass (g)	Std. deviation
DK	462.90	203.911
FLK Zinj	351.36	160.68
FLK North 1-2	390.56	151.267
Experimental selection	378.24	102.04

Table S4. Innovation of toolmaking behavior.

Individual	Naivety	Did participant make a cutting tool? (y/n)	Did participant make *and* use a cutting tool? (y/n)	First potential toolmaking event				First confirmed toolmaking event				*n* of successful solutions (i.e. opening tendon box)
				Mins. elapsed	*n* of prior solution attempts	Trial #	TM Teq.	Mins. elapsed	*n* of prior solution attempts	Trial #	TM Teq.	
P1	3	Y	Y	1	0	1	PH	1	0	1	PH	35
P2	2	Y	Y	5	7	3	FH	29	8	4	PH	4
P3	2	Y	Y	2	0	1	BP	2	0	1	BP	54
P4	2	Y	Y	14	2	3	FH	37	12	5	PH	51
P5	2	Y	Y	3	2	1	PJ	3	2	1	PJ	73
P6	2	Y	Y	98	56	2	FH	238	75	6	PH	6
P7	1	Y	Y	7	4	2	PJ	25	16	4	BP	55
P8	2	Y	Y	1	1	1	PH	1	1	1	PH	70
P9	2	Y	Y	9	1	2	FH	9	1	2	FH	60
P10	2	Y	Y	26	15	5	FH	31	16	6	BP	38
P11	0	Y	Y	31	9	4	BP	31	9	4	BP	57
P12	2	Y	Y	1	1	1	FH	10	7	2	FH	72
P13	2	Y	Y	17	15	3	PH	20	15	3	FH	52
P14	0	Y	Y	21	10	1	FH	27	11	2	FH	57
P15	2	Y	Y	17	15	3	BP	17	16	3	BP	54
P16	2	Y	Y	70	29	6	FH	77	31	8	FH	45
P17	2	Y	Y	4	6	1	PH	4	6	1	PH	55
P19	4	Y	Y	10	5	2	PH	26	28	2	BP	55
P21	2	Y	N	79	69	4	PJ	194	115	7	BP	7
P22	2	Y	Y	8	2	2	BP	8	2	2	BP	27
P23	2	Y	N	17	17	1	BP	n.a.	n.a.	n.a.	n.a.	7
P24	2	Y	Y	32	19	3	FH	32	19	3	FH	70
P25	2	Y	Y	45	3	1	PH	47	31	3	PH	57
P26	2	Y	Y	6	5	2	BP	6	5	2	BP	59
P27	2	Y	Y	2	1	1	FH	3	1	1	FH	53
P28	3	Y	Y	1	0	1	PH	1	0	1	PH	64
P29	2	Y	Y	41	31	4	PJ	82	53	6	FH	59
P30	2	Y	Y	10	2	3	BP	10	2	3	BP	78
			Mean	20.6 ±25.2	11.7 ±16.7	2.3 ±1.4	n.a.	36.0 ±56.3	17.9 ±26.1	3.1 ±2.0	n.a.	49.1 ±21.1

Table S5. Technique of first potential and confirmed toolmaking events.

Event	Passive Hammer		Bipolar		Freehand		Projectile	
	n	%	*n*	%	*n*	%	*n*	%
First potential toolmaking event	7	25.0%	7	25.0%	10	35.7%	4	14.3%
First confirmed toolmaking event	8	29.6%	10	37.0%	8	29.6%	1	3.7%

Table S6. Preferences for techniques in potential and confirmed toolmaking.

Condition	Passive Hammer		Bipolar		Freehand		Projectile		Anvil-oriented		Hand-oriented		Opportunistic	
	n	%	*n*	%	*n*	%	*n*	%	*n*	%	*n*	%	*n*	%
Potential TM events	7	25.0	10	35.7	4	14.3	0	0	5	17.9	0	0	2	7.1
TM events with observable fracture	10	37.0	13	48.1	3	11.1	0	0	0	0	0	0	1	3.7

Table S7. Distribution of techniques for potential toolmaking events.

Individual	*N* of TM	Passive Hammer		Bipolar		Freehand		Projectile		Preference
		n	%	*n*	%	*n*	%	*n*	%	
P1	41	41	92.7	3	7.3	0	0	0	0	PH
P2	9	4	44.4	1	11.1	1	11.1	3	33.3	AO
P3	50	3	6.0	47	94.0	0	0	0	0	BP
P4	59	36	61.0	14	23.7	8	13.6	1	1.7	PH
P5	41	40	97.6	0	0	0	0	1	2.4	PH
P6	25	5	20.0	3	12.0	17	68.0	0	0	FH
P7	57	24	42.1	4	7.0	28	49.1	1	1.8	OP
P8	44	1	2.3	2	4.5	41	93.2	0	0	FH
P9	62	4	6.5	32	51.6	26	41.9	0	0	BP
P10	34	3	8.8	25	73.5	5	14.7	1	2.9	BP
P11	38	1	2.6	36	94.7	1	2.6	0	0	BP
P12	34	0	0	32	94.1	2	5.9	0	0	BP
P13	74	16	21.6	54	73.0	4	5.4	0	0	BP
P14	58	54	93.1	1	1.7	3	5.2	0	0	PH
P15	87	26	29.9	42	48.3	7	8.0	12	13.8	AO
P16	47	21	44.7	20	42.6	6	12.8	0	0	AO
P17	73	57	78.1	9	12.3	7	9.6	0	0	PH
P19	56	2	3.6	54	96.4	0	0	0	0	BP
P21	25	2	8.0	12	48.0	0	0	11	44.0	AO
P22	153	72	47.1	55	35.9	26	17.0	0	0	AO
P23	16	0	0	16	100.0	0	0	0	0	BP
P24	46	0	0	23	50.0	23	50.0	0	0	OP
P25	52	52	100.0	0	0	0	0	0	0	PH
P26	77	67	87.0	3	3.9	4	5.2	3	3.9	PH
P27	65	11	16.9	33	50.8	21	32.3	0	0	BP
P28	68	20	29.4	48	70.6	0	0	0	0	BP
P29	47	0	0	0	0	44	93.6	3	6.4	FH
P30	142	0	0	5	3.5	136	95.8	1	0.7	FH
Overall	1580	559	35.4	574	36.3	410	25.9	37	2.3	
Mean	56.429	20.071	n.a.	20.5	n.a.	14.643	n.a.	1.321	n.a.	
Std. dev.	31.635	23.090	n.a.	19.346	n.a.	27.063	n.a.	3.031	n.a.	

Table S8. Frequency of coded potential toolmaking (TM) events. *%* is the percentage of total bouts represented by a category. κ_1 is the Cohen's kappa coefficient calculated using exact counts of the relevant bouts per trial. κ_2 is the Cohen's kappa coefficient calculated using Boolean data (presence/absence of the specific bout per trial). Where fracture and severing of the puzzle box rope occurred simultaneously, the event was coded as toolmaking and use (TMU).

	n	%	$\bar{x}$	x_{min}	x_{max}	κ_1	κ_2
TM bouts	1580	100.0	56.4	9 (P2)	153 (P22)	0.707	0.978
TMU bouts	260	16.5	9.3	0	79 (P30)	0.538	0.721
Freehand	410	25.9	14.6	0	136 (P30)	0.632	0.939
Passive hammer	559	35.4	20.1	0	72 (P22)	0.756	0.991
Bipolar	574	36.3	20.5	0	55 (P22)	0.932	0.966
Projectile	37	2.3	1.3	0	12 (P15)	1.000	1.000

Table S9. Distribution of techniques for confirmed toolmaking events.

Individual	*N* of TM	Passive Hammer		Bipolar		Freehand		Projectile		Preference
		n	%	*n*	%	*n*	%	*n*	%	
P1	39	37	94.9	2	5.1	0	0	0	0	PH
P2	5	4	80.0	1	20.0	0	0	0	0	PH
P3	49	3	6.1	46	93.9	0	0	0	0	BP
P4	35	26	74.3	9	25.7	0	0	0	0	PH
P5	40	39	97.5	0	0	0	0	1	2.5	PH
P6	2	2	100.0	0	0	0	0	0	0	PH
P7	36	17	47.2	2	5.6	17	47.2	0	0	OP
P8	41	1	2.4	1	2.4	39	95.1	0	0	FH
P9	49	2	4.1	32	65.3	15	30.6	0	0	BP
P10	23	0	0	22	95.7	1	4.3	0	0	BP
P11	36	0	0	36	100.0	0	0	0	0	BP
P12	33	0	0	32	97.0	1	3.0	0	0	BP
P13	56	6	10.7	49	87.5	1	1.8	0	0	BP
P14	54	53	98.1	0	0	1	1.9	0	0	PH
P15	58	17	29.3	31	53.4	6	10.3	4	6.9	BP
P16	28	7	25.0	17	60.7	4	14.3	0	0	BP
P17	57	47	82.5	8	14.0	2	3.5	0	0	PH
P19	50	0	0	50	100.0	0	0	0	0	BP
P21	6	1	16.7	5	83.3	0	0	0	0	BP
P22	49	28	57.1	19	38.8	2	4.1	0	0	PH
P24	31	0	0	22	71.0	9	29.0	0	0	BP
P25	46	46	100.0	0	0	0	0	0	0	PH
P26	45	39	86.7	3	6.7	3	6.7	0	0	PH
P27	46	5	10.9	29	63.0	12	26.1	0	0	BP
P28	66	18	27.3	48	72.7	0	0	0	0	BP
P29	43	0	0	0	0	43	100.0	0	0	FH
P30	72	0	0	2	2.8	70	97.2	0	0	FH
Overall	1095	398	36.3	466	42.6	226	20.6	5	0.5	
Mean	40.6	19.9	n.a.	21.2	n.a.	14.1	n.a.	2.5	n.a.	
Std. dev.	17.2	18.0	n.a.	17.5	n.a.	19.8	n.a.	2.1	n.a.	

Table S10. Artefact type distributions for the assemblages.

		Total	Flakes		Angular Fragments		Glass Cores		River Cobbles	
			n	%	*n*	%	*n*	%	*n*	%
Individual	P1	39	26	66.7%	11	28.2%	1	2.6%	1	2.6%
	P2	3	0	0.0%	1	33.3%	1	33.3%	1	33.3%
	P3	114	76	66.7%	36	31.6%	1	0.9%	1	0.9%
	P4	43	35	81.4%	6	14.0%	1	2.3%	1	2.3%
	P5	38	23	60.5%	13	34.2%	1	2.6%	1	2.6%
	P6	5	2	40.0%	1	20.0%	1	20.0%	1	20.0%
	P7	41	26	63.4%	13	31.7%	1	2.4%	1	2.4%
	P8	76	45	59.2%	29	38.2%	1	1.3%	1	1.3%
	P9	64	46	71.9%	16	25.0%	1	1.6%	1	1.6%
	P10	45	35	77.8%	8	17.8%	1	2.2%	1	2.2%
	P11	41	35	85.4%	4	9.8%	1	2.4%	1	2.4%
	P12	67	53	79.1%	11	16.4%	2	3.0%	1	1.5%
	P13	104	85	81.7%	17	16.3%	1	1.0%	1	1.0%
	P14	77	61	79.2%	14	18.2%	1	1.3%	1	1.3%
	P15	75	42	56.0%	30	40.0%	1	1.3%	2	2.7%
	P16	83	43	51.8%	37	44.6%	1	1.2%	2	2.4%
	P17	124	105	84.7%	15	12.1%	3	2.4%	1	0.8%
	P19	95	77	81.1%	14	14.7%	3	3.2%	1	1.1%
	P21	8	2	25.0%	4	50.0%	1	12.5%	1	12.5%
	P22	80	63	78.8%	14	17.5%	1	1.3%	2	2.5%
	P23	4	0	0.0%	2	50.0%	1	25.0%	1	25.0%
	P24	41	37	90.2%	2	4.9%	1	2.4%	1	2.4%
	P25	60	48	80.0%	10	16.7%	1	1.7%	1	1.7%
	P26	30	26	86.7%	2	6.7%	1	3.3%	1	3.3%
	P27	45	39	86.7%	4	8.9%	1	2.2%	1	2.2%
	P28	108	60	55.6%	44	40.7%	1	0.9%	3	2.8%
	P29	57	53	93.0%	2	3.5%	1	1.8%	1	1.8%
	P30	32	29	90.6%	1	3.1%	1	3.1%	1	3.1%
	Mean	57.1±33.6	41.9±25.7		12.9±12.0		1.2±0.5		1.2±0.5	
Naivety Level	0	118	96	81.4%	18	15.3%	2	1.7%	2	1.7%
	1	41	26	63.4%	13	31.7%	1	2.4%	1	2.4%
	2	1198	887	74.0%	261	21.8%	25	2.1%	25	2.1%
	3	147	86	58.5%	55	37.4%	2	1.4%	4	2.7%
	4	95	77	81.1%	14	14.7%	3	3.2%	1	1.1%
**Total**		1599	1172	73.3%	361	22.6%	33	2.1%	33	2.1%

Table S11. p-Values from pairwise comparisons of PD using Wilcoxon rank sum tests. Cells for statistically significant comparisons marked in grey.

**Naivety Level**	2	3&4	Oldowan (*46*)
0&1	0.14559	0.28869	0.00011
2		0.61145	$< 2 \times 10^{-16}$
3&4			2.1×10^{-9}

Table S12. p-Values from pairwise comparisons of EPA using Wilcoxon rank sum tests.

**Naivety Level**	2	3&4	Oldowan (*46*)
0&1	0.72	0.63	0.83
2		0.87	0.59
3&4			0.64

Movie S1.

This file contains video clips (.mp4) taken from several trials in the study, displaying some of the early knapping techniques innovated by the participants to create and use cutting tools.

Movie S2.

This file contains video (.mp4) of rotating 3D scans of the original blank form (the painted glass hemisphere) and of the two knapped cores produced by totally naïve participants P11 (Example 1, artefact ID: Z11.C16.36) and P14 (Example 2, artefact ID: Z14.C19.55).

Data S1.

This file contains the primary data (.xlsx) for metric attributes, including platform depth and exterior platform angle, of experimental and archaeological (*46*) flakes.

REFERENCES AND NOTES

1. J. Henrich, *The Secret of Our Success: How Culture Is Driving Human Evolution, Domesticating Our Species, and Making Us Smarter* (Princeton University Press, 2016).

2. R. Boyd, *A Different Kind of Animal: How Culture Transformed Our Species* (Princeton Univ. Press, 2018).

3. C. Tennie, E. Bandini, C. P. van Schaik, L. M. Hopper, The zone of latent solutions and its relevance to understanding ape cultures. *Biol. Philos.* **35**, 55 (2020).

4. R. Boyd, P. J. Richerson, Why culture is common, but cultural evolution is rare. *Proc. Br. Acad.* **88**, 77–93 (1996).

5. E. Bandini, A. Motes-Rodrigo, M. P. Steele, C. Rutz, C. Tennie, Examining the mechanisms underlying the acquisition of animal tool behaviour. *Biol. Lett.* **16**, 20200122 (2020).

6. E. Reindl, S. R. Beck, I. A. Apperly, C. Tennie, Young children spontaneously invent wild great apes' tool-use behaviours. *Proc. R. Soc. B* **283**, 20152402 (2016).

7. K. D. Schick, N. Toth, *Making Silent Stones Speak: Human Evolution and the Dawn of Technology* (Simon & Schuster, 1994).

8. M. V. Caruana, F. d'Errico, L. Backwell, Early hominin social learning strategies underlying the use and production of bone and stone tools, in *Tool Use in Animals*, C. Sanz, J. Call, C. Boesch, Eds. (Cambridge University Press, 2013), pp. 242–285.

9. C. Shipton, M. Nielsen, Before cumulative culture: The evolutionary origins of overimitation and shared intentionality. *Hum. Nat.* **26**, 331–345 (2015).

10. T. J. H. Morgan, N. T. Uomini, L. E. Rendell, L. Chouinard-Thuly, S. E. Street, H. M. Lewis, C. P. Cross, C. Evans, R. Kearney, I. de la Torre, A. Whiten, K. N. Laland, Experimental evidence for the co-evolution of hominin tool-making teaching and language. *Nat. Commun.* **6**, 6029 (2015).

11. D. Stout, M. J. Rogers, A. V. Jaeggi, S. Semaw, Archaeology and the origins of human cumulative culture: A case study from the earliest Oldowan at Gona, Ethiopia. *Curr. Anthropol.* **60**, 309–340 (2019).

12. S. H. Ambrose, Paleolithic technology and human evolution. *Science* **291**, 1748–1753 (2001).

13. D. R. Braun, V. Aldeias, W. Archer, J. R. Arrowsmith, N. Baraki, C. J. Campisano, A. L. Deino, E. N. DiMaggio, G. Dupont-Nivet, B. Engda, D. A. Feary, D. I. Garello, Z. Kerfelew, S. P. McPherron, D. B. Patterson, J. S. Reeves, J. C. Thompson, K. E. Reed, Earliest known Oldowan artifacts at >2.58 Ma from Ledi-Geraru, Ethiopia, highlight early technological diversity. *Proc. Natl. Acad. Sci. U.S.A.* **116**, 11712–11717 (2019).

14. N. Toth, The Oldowan reassessed: A close look at early stone artifacts. *J. Archaeol. Sci.* **12**, 101–120 (1985).

15. B. Cotterell, J. Kamminga, The formation of flakes. *Am. Antiq.* **52**, 675–708 (1987).

16. T. Wynn, W. C. McGrew, An ape's view of the Oldowan. *Man* **24**, 383 (1989).

17. G. Clark, *World Prehistory: A New Outline* (Cambridge Univ. Press, 1969).

18. J. J. Shea, Lithic Modes A–I: A new framework for describing global-scale variation in stone tool technology illustrated with evidence from the East Mediterranean Levant. *J. Archaeol. Method Theory* **20**, 151–186 (2013).

19. L. Harlacker, Knowledge and know-how in the Oldowan: An experimental approach, in *Skilled Production and Social Reproduction*, J. Apel, K. Knutsson, Eds. (Societas Archaeologica Upsaliensis, 2003), pp. 219–243.

20. D. Stout, E. Hecht, N. Khreisheh, B. Bradley, T. Chaminade, Cognitive demands of Lower Paleolithic toolmaking. *PLOS ONE* **10**, 0121804 (2015).

21. D. Stout, T. Chaminade, J. Apel, A. Shafti, A. A. Faisal, The measurement, evolution, and neural representation of action grammars of human behavior. *Sci. Rep.* **11**, 13720 (2021).

22. D. Lombao, M. Guardiola, M. Mosquera, Teaching to make stone tools: New experimental evidence supporting a technological hypothesis for the origins of language. *Sci. Rep.* **7**, 14394 (2017).

23. D. M. Cataldo, A. B. Migliano, L. Vinicius, Speech, stone tool-making and the evolution of language. *PLOS ONE* **13**, 0191071 (2018).

24. J. Pargeter, S. Dietrich, L. Cheng, M. B. Kilgore, Testing the Social, Cognitive, and Motor Foundations of Paleolithic Skill Reproduction (OSF, 2021), doi:10.17605/OSF.IO/YEBRU [31 August 2021].

25. E. Reindl, I. A. Apperly, S. R. Beck, C. Tennie, Young children copy cumulative technological design in the absence of action information. *Sci. Rep.* **7**, 1788 (2017).

26. S. S. Putt, The origins of stone tool reduction and the transition to knapping: An experimental approach. *J. Archaeol. Sci. Rep.* **2**, 51–60 (2015).

27. D. Stout, Stone toolmaking and the evolution of human culture and cognition. *Phil. Trans. R. Soc. B.* **366**, 1050–1059 (2011).

28. D. Stout, R. Passingham, C. Frith, J. Apel, T. Chaminade, Technology, expertise and social cognition in human evolution: Technology and cognition in human evolution. *Eur. J. Neurosci.* **33**, 1328–1338 (2011).

29. M. S. Bisson, Interview with a Neanderthal: An experimental approach for reconstructing scraper production rules, and their implications for imposed form in Middle Palaeolithic tools. *Cambridge Archaeol. J.* **11**, 165–184 (2001).

30. D. Stout, S. Semaw, Understanding Oldowan knapping skill: An experimental study of skill acquisition in modern humans, in *The Oldowan: Case Studies into the Earliest Stone Age*, N. Toth, K. Schick, Eds. (Stone Age Institute Press, 2006), pp. 307–320.

31. D. Stout, K. Schick, N. Toth, Knapping skill of the earliest stone toolmakers: Insights from the study of modern human novices, in *The Cutting Edge: New Approaches in the Archaeology of Human Origins*, N. Toth, K. Schick, Eds. (Stone Age Institute Press, 2009), pp. 247–266.

32. R. V. S. Wright, Imitative learning of a flaked stone technology—The case of an orangutan. *Mankind.* **8**, 296–306 (1972).

33. S. S. Putt, A. D. Woods, R. G. Franciscus, The role of verbal interaction during experimental bifacial stone tool manufacture. *Lithic Technol.* **39**, 96–112 (2014).

34. C. Tennie, D. R. Braun, L. S. Premo, S. P. McPherron, The island test for cumulative culture in the Paleolithic, in *The Nature of Culture*, M. N. Haidle, N. J. Conard, M. Bolus, Eds. (Vertebrate Paleobiology and Paleoanthropology, Springer, 2016), pp. 121–133.

35. C. Tennie, L. S. Premo, D. R. Braun, S. P. McPherron, Early stone tools and cultural transmission: Resetting the null hypothesis. *Curr. Anthropol.* **58**, 652–672 (2017).

36. E. Bandini, C. Tennie, Spontaneous reoccurrence of "scooping", a wild tool-use behaviour, in naïve chimpanzees. *PeerJ.* **5**, e3814 (2017).

37. D. Neadle, M. Allritz, C. Tennie, Food cleaning in gorillas: Social learning is a possibility but not a necessity. *PLOS ONE* **12**, e0188866 (2017).

38. M. Tomasello, The human adaptation for culture. *Ann. Rev. Anthropol.* **28**, 509–529 (1999).

39. N. Toth, K. D. Schick, E. S. Savage-Rumbaugh, R. A. Sevcik, D. M. Rumbaugh, Pan the Tool-Maker: Investigations into the stone tool-making and tool-using capabilities of a bonobo (*Pan paniscus*). *J. Archaeol. Sci.* **20**, 81–91 (1993).

40. E. Bandini, A. Motes-Rodrigo, W. Archer, T. Minchin, H. Axelsen, R. A. Hernandez-Aguilar, S. P. McPherron, C. Tennie, Naïve, unenculturated chimpanzees fail to make and use flaked stone tools. *Open Res. Europe.* **1**, 20 (2021).

41. A. Motes-Rodrigo, S. P. McPherron, W. Archer, R. A. Hernandez-Aguilar, C. Tennie, Experimental investigation of orangutans' lithic percussive and sharp stone tool behaviours. *PLOS ONE* **16**, e0263343 (2022).

42. N. Toth, K. Schick, Factors affecting variability in Early Stone Age lithic assemblages: Personal observations from actualistic studies, in *Casting the Net Wide: Papers in Honor of Glynn Isaac and His Approach to Human Origins Research*, J. Sept, D. Pilbeam, Eds. (Oxbow Books, 2011), pp. 53–74.

43. N. Toth, K. Schick, An overview of the cognitive implications of the Oldowan industrial complex. *Azania.* **53**, 3–39 (2018).

44. S. Harmand, J. E. Lewis, C. S. Feibel, C. J. Lepre, S. Prat, A. Lenoble, X. Boës, R. L. Quinn, M. Brenet, A. Arroyo, N. Taylor, S. Clément, G. Daver, J.-P. Brugal, L. Leakey, R. A. Mortlock, J. D. Wright, S. Lokorodi, C. Kirwa, D. V. Kent, H. Roche, 3.3-million-year-old stone tools from Lomekwi 3, West Turkana, Kenya. *Nature* **521**, 310–315 (2015).

45. I. de la Torre, Searching for the emergence of stone tool making in eastern Africa. *Proc. Natl. Acad. Sci. USA* **116**, 11567–11569 (2019).

46. Ž. Režek, H. L. Dibble, S. P. McPherron, D. R. Braun, S. C. Lin, Two million years of flaking stone and the evolutionary efficiency of stone tool technology. *Nat. Ecol. Evol.* **2**, 628–633 (2018).

47. J. W. K. Harris, G. Isaac, The Karari Industry: Early Pleistocene archaeological evidence from the terrain east of Lake Turkana, Kenya. *Nature* **262**, 102–107 (1976).

48. H. Duke, J. Pargeter, Weaving simple solutions to complex problems: An experimental study of skill in bipolar cobble-splitting. *Lithic Technol.* **40**, 349–365 (2015).

49. J. R. Ferguson, An experimental test of the conservation of raw material in flintknapping skill acquisition. *Lithic Technol.* **28**, 113–131 (2003).

50. F. Sternke, M. Sørensen, The identification of children's flintknapping products in Mesolithic Scandinavia, in *Mesolithic Horizons. Papers Presented at the Seventh International Conference on the Mesolithic in Europe, Belfast 2005*, S. McCartan, R. Schulting, G. Warren, P. C. Woodman, Eds. (Oxbow Books, 2007).

51. P. Horta, N. Bicho, J. Cascalheira, Lithic bipolar methods as an adaptive strategy through space and time. *J. Archaeol. Sci. Rep.* **41**, 103263 (2022).

52. M. W. Moore, Y. Perston, Experimental insights into the cognitive significance of early stone tools. *PLOS ONE* **11**, e0158803 (2016).

53. M. I. Eren, S. J. Lycett, R. J. Patten, B. Buchanan, J. Pargeter, M. J. O'Brien, Test, model, and method validation: The role of experimental stone artifact replication in hypothesis-driven archaeology. *Ethnoarchaeology* **8**, 103–136 (2016).

54. S. C. Lin, Z. Rezek, H. L. Dibble, Experimental design and experimental inference in stone artifact archaeology. *J. Archaeol. Method Theory* **25**, 663–688 (2018).

55. M. Piperno, C. Collina, R. Gallotti, J.-P. Raynal, G. Kieffer, F.-X. le Bourdonnec, G. Poupeau, D. Geraads, Obsidian exploitation and utilization during the Oldowan at Melka Kunture (Ethiopia), in *Interdisciplinary Approaches to the Oldowan*, E. Hovers, D. R. Braun, Eds. (Vertebrate Paleobiology and Paleoanthropology, Springer, 2009), pp. 111–128.

56. T. Dogandžić, A. Abdolazadeh, G. Leader, L. Li, S. P. McPherron, C. Tennie, H. L. Dibble, The results of lithic experiments performed on glass cores are applicable to other raw materials. *Archaeol. Anthropol. Sci.* **12**, 44 (2020).

57. T. Nonaka, B. Bril, R. Rein, How do stone knappers predict and control the outcome of flaking? Implications for understanding early stone tool technology. *J. Hum. Evol.* **59**, 155–167 (2010).

58. D. Stout, T. Chaminade, The evolutionary neuroscience of tool making. *Neuropsychologia* **45**, 1091–1100 (2007).

59. E. Hovers, Invention, Reinvention and Innovation: The makings of Oldowan Lithic Technology, in *Developments in Quaternary Sciences*, S. Elias, Ed. (Elsevier, 2012), vol. 16, pp. 51–68.

60. J. S. Reeves, D. R. Braun, E. Finestone, T. W. Plummer, Ecological perspectives on technological diversity at Kanjera South. *J. Hum. Evol.* **158**, 103029 (2021).

61. M. N. Haidle, Working-memory capacity and the evolution of modern cognitive potential: Implications from animal and early human tool use. *Curr. Anthropol.* **51**, S149–S166 (2010).

62. G. R. Pradhan, C. Tennie, C. P. van Schaik, Social organization and the evolution of cumulative technology in apes and hominins. *J. Hum. Evol.* **63**, 180–190 (2012).

63. D. Stout, J. Apel, J. Commander, M. Roberts, Late Acheulean technology and cognition at Boxgrove, UK. *J. Archaeol. Sci.* **41**, 576–590 (2014).

64. S. S. Putt, S. Wijeakumar, R. G. Fraciscus, J. P. Spencer, The functional brain networks that underlie Early Stone Age tool manufacture. *Nat. Hum. Behav.* **1**, 0102 (2017).

65. S. S. Putt, S. Wijeakumar, J. P. Spencer, Prefrontal cortex activation supports the emergence of early stone age toolmaking skill. *Neuroimage* **199**, 57–69 (2019).

66. D. W. Read, H. M. Manrique, M. J. Walker, On the working memory of humans and great apes: Strikingly similar or remarkably different? *Neurosci. Biobehav. R.* **134**, 104496 (2022).

67. J. Henrich, S. J. Heine, A. Norenzayan, The weirdest people in the world? *Behav. Brain Sci.* **33**, 61–83 (2010).

68. I. de la Torre, R. Mora, *Technological Strategies in the Lower Pleistocene at Olduvai Beds I & II.* (Etudes et Recherches Archeologiques de l'Universite de Liege, 2005).

69. A. J. M. Key, S. J. Lycett, Are bigger flakes always better? An experimental assessment of flake size variation on cutting efficiency and loading. *J. Archaeol. Sci.* **41**, 140–146 (2014).

70. I. de la Torre, The Early Stone Age lithic assemblages of Gadeb (Ethiopia) and the developed Oldowan/early Acheulean in East Africa. *J. Hum. Evol.* **60**, 768–812 (2011).

71. M. D. Leakey, *Olduvai Gorge, Volume 3: Excavations in Beds I and II, 1960–1963* (Cambridge Univ. Press, 1971).

72. RStudio Team, RStudio: Integrated Development for R (2020); http://rstudio.com/.

73. S. Semaw, The oldest stone artifacts from Gona (2.6–2.5 Ma), Afar, Ethiopia: Implications for understanding the earliest stages of stone knapping, in *The Oldowan: Case Studies into the Earliest Stone Age*, N. Toth, K. Schick, Eds. (Stone Age Institute Press, 2006), pp. 307–320.

74. W. H. Kimbel, R. C. Walter, D. C. Johanson, K. E. Reed, J. L. Aronson, Z. Assefa, C. W. Marean, G. G. Eck, R. Bobe, E. Hovers, Y. Rak, C. Vondra, T. Yemane, D. York, Y. Chen, N. M. Evensen, P. E. Smith, Late Pliocene *Homo* and Oldowan tools from the Hadar formation (Kada Hadar Member), Ethiopia. *J. Hum. Evol.* **31**, 549–561 (1996).

75. M. Tomasello, *The Cultural Origins of Human Cognition* (Harvard Univ. Press, 4. print., 2003).

76. C. Tennie, J. Call, M. Tomasello, Ratcheting up the ratchet: On the evolution of cumulative culture. *Phil. Trans. R. Soc. B.* **364**, 2405–2415 (2009).

77. D. B. Bamforth, N. Finlay, Introduction: Archaeological approaches to lithic production skill and craft learning. *J. Archaeol. Method Theory* **15**, 1–27 (2008).

78. J. Pargeter, N. Khreisheh, J. J. Shea, D. Stout, Knowledge vs know-how?, Dissecting the foundations of stone knapping skill. *J. Hum. Evol.* **145**, 102807 (2020).

79. J. N. Cerasoni, Vectorial application for the illustration of archaeological lithic artefacts using the “Stone Tools Illustrations with Vector Art” (STIVA) Method. *PLOS ONE* **16**, 0251466 (2021).
